# Loss of genes for DNA recombination and repair in the reductive genome evolution of thioautotrophic symbionts of *Calyptogena *clams

**DOI:** 10.1186/1471-2148-11-285

**Published:** 2011-10-03

**Authors:** Hirokazu Kuwahara, Yoshihiro Takaki, Shigeru Shimamura, Takao Yoshida, Taro Maeda, Takekazu Kunieda, Tadashi Maruyama

**Affiliations:** 1 Marine Biodiversity Research Program, Japan Agency for Marine-Earth Science and Technology, Natsushima-cho, Yokosuka, Kanagawa 237-0061, Japan; 2 Department of Biological Sciences, Graduate School of Science, The University of Tokyo, Hongo, Bunkyo-ku, Tokyo 113-0033, Japan; 3 Graduate School of Marine Science and Technology, Tokyo University of Marine Science and Technology, Konan, Minato-ku, Tokyo 108-8477, Japan

## Abstract

**Background:**

Two *Calyptogena *clam intracellular obligate symbionts, *Ca*. Vesicomyosocius okutanii (Vok; *C. okutanii *symbiont) and *Ca*. Ruthia magnifica (Rma; *C. magnifica *symbiont), have small genomes (1.02 and 1.16 Mb, respectively) with low G+C contents (31.6% and 34.0%, respectively) and are thought to be in an ongoing stage of reductive genome evolution (RGE). They lack *recA *and some genes for DNA repair, including *mutY*. The loss of *recA *and *mutY *is thought to contribute to the stabilization of their genome architectures and GC bias, respectively. To understand how these genes were lost from the symbiont genomes, we surveyed these genes in the genomes from 10 other *Calyptogena *clam symbionts using the polymerase chain reaction (PCR).

**Results:**

Phylogenetic trees reconstructed using concatenated 16S and 23S rRNA gene sequences showed that the symbionts formed two clades, clade I (symbionts of *C. kawamurai*, *C. laubieri*, *C. kilmeri*, *C. okutanii and C. soyoae*) and clade II (those of *C. pacifica*, *C. fausta*, *C. nautilei*, *C. stearnsii*, *C. magnifica*, *C. fossajaponica and C. phaseoliformis*). *recA *was detected by PCR with consensus primers for *recA *in the symbiont of *C. phaseoliformis*. A detailed homology search revealed a remnant *recA *in the Rma genome. Using PCR with a newly designed primer set, intact *recA *or its remnant was detected in clade II symbionts. In clade I symbionts, the *recA *coding region was found to be mostly deleted.

In the Rma genome, a pseudogene of *mutY *was found. Using PCR with newly designed primer sets, *mutY *was not found in clade I symbionts but was found in clade II symbionts. The G+C content of 16S and 23S rRNA genes in symbionts lacking *mutY *was significantly lower than in those with *mutY*.

**Conclusions:**

The extant *Calyptogena *clam symbionts in clade II were shown to have *recA *and *mutY *or their remnants, while those in clade I did not. The present results indicate that the extant symbionts are losing these genes in RGE, and that the loss of *mutY *contributed to the GC bias of the genomes during their evolution.

## Background

Recent genome analyses have shown that the genomes of intracellular obligate symbionts, which are vertically transmitted to the next generation of their hosts, have a tendency to reduce in size during evolution [1-3]. Generally, smaller genomes have a lower G+C content (GC bias) [3], although there are some exceptions, e.g., a symbiont of cicadas, *Ca*. Hodgkinia cicadicola has a very small genome (150 kb) with a relatively high G+C content (58.4%) [4]. Genes for DNA recombination and repair, i.e., *recA *and *uvrA to C*, are deleted in many intracellular symbiont genomes [1,5,6].

Reductive genome evolution (RGE) has been extensively studied in insect symbionts. *Buchnera *strains, which are intracellular heterotrophic symbionts of aphids, have small genomes (0.45-0.65 Mb). Although the genome architectures of the extant *Buchnera *strains are stable, RGE is ongoing with small deletions at a slower rate than in its early stage [7-9]. However, the earlier stages of RGE are still largely unknown.

Intracellular symbiotic chemoautotrophic bacteria are ubiquitous in deep-sea invertebrates such as *Calyptogena *clams [10]. *Calyptogena *clam symbionts are thought to be vertically transmitted via eggs [11,12]. The similarity between the phylogenetic topologies of *Calyptogena *clams and their symbionts indicates their co-evolution [13], although the possibility of lateral acquisition of the symbionts in some *Calyptogena *clams has been reported [14,15]. The genomes of symbionts in *Calyptogena magnifica *(*Ca*. Ruthia magnifica, Rma, 1.16 Mb) and in *C. okutanii *(*Ca*. Vesicomyosocius okutanii, Vok, 1.02 Mb) have been reported [16,17]. They lack large-sized repeated sequences (> 200 bp), phage and mobile genetic elements [16,17]. A comparative analysis of these genomes showed that the RGE in *Calyptogena *symbiont genomes is currently ongoing and is still in an earlier stage than that in the *Buchnera *strains [8]. Further, it has been reported that both of the *Calyptogena *symbionts lack genes for DNA recombination and repair such as *recA *and *mutY *[17].

Recombinase RecA is a key enzyme for homologous recombination [18]. It requires relatively long repeated sequences (> 200 bp) for recombination [19] and is a possible driving mechanism of dynamic genome rearrangement including large deletions. In RGE in symbionts of *Calyptogena *clams, RecA probably functioned to delete large gene sequences by recombination, consuming the long repeated sequences in the early stage of RGE [8]. On the other hand, MutY is known to repair A-G mispairs to C-G pairs [20]. Thus, the loss of *mutY *is thought to cause decreasing G+C content of the genome.

While losses of genes for DNA repair and recombination may occur spontaneously, they affect the later stage of RGE by increasing mutation rates, affecting the GC bias and regenerating short repeated sequences [8]. After the loss of *recA*, the genome architecture probably stabilized in the clam symbionts [8]. In the insect symbiont *Buchnera*, *recA *was reported to be lost in its early evolution [21]. The contribution of RecA to RGE in intracellular symbionts is still controversial. It was shown that small deletions with a size of up to 200 kb occur without recognizable repeats via RecA-independent recombination events in *Salmonella *[22]. To understand the roles of RecA-dependent and -independent recombination events in RGE in intracellular symbiosis, it is important to determine when and how *recA *was lost in their lineages and the effects of its loss on their RGE. However, little is known about the relationship between the loss of DNA repair/recombination genes and RGE. To understand the effects of their loss on RGE, we posed the question of whether these genes had been lost before the divergence of the *Calyptogena *clam symbionts or whether they remained in some symbionts thereafter. To address this question, we searched for *recA*, *mutY *and/or their remnants in the genomes of 10 *Calyptogena *clam symbionts in addition to Rma and Vok.

## Results

### Phylogenetic relationships of *Calyptogena *clam symbionts

**Figure 1:**
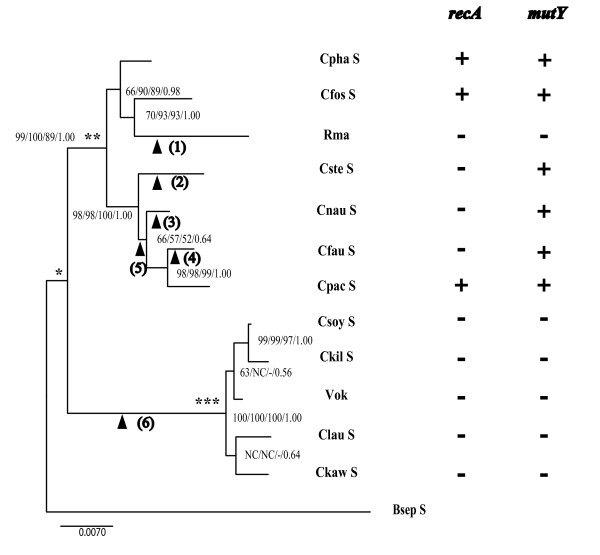
**Phylogenetic tree of the *Calyptogena *clam symbionts with a table showing the presence/absence of *mutY *and *recA***. The maximum likelihood tree based on concatenated 16S and 23S rDNA sequences is shown with bootstrap values (> 50%) obtained from the neighbor joining, maximum parsimony and maximum likelihood methods, and with the posterior probabilities obtained in the Bayesian method at each node. The table shows the presence (+) and absence (-) of functional *mutY *and *recA*. Abbreviations of the symbionts and accession numbers of the sequences are shown in Table 3. Black triangle (1), Inactivation of *recA *by deletions (see Figure 2B and Additional file 1, Figure S1), and a substitution mutation of G to A at position 568 to truncate *mutY *and deletions at positions 797-799 and 974-989 of *mutY *in Rma (see Additional file 2, Figure S2). Black triangle (2), Inactivation of *recA *by substitution and deletion mutations in the *C. stearnsii *symbiont. Black triangle (3), Inactivation of *recA *by a two-base insertion of "CC" at position 483-484, making a new stop codon, in the *C. nautilei *symbiont (see Additional file 1, Figure S1). Black triangle (4), Inactivation of *recA *by a two-base insertion of "CC" at position 483-484, making a new stop codon, in the *C. fausta *symbiont lineage (see Additional file 1, Figure S1). Black triangle (5), A deletion of almost the same size outside the *recA *coding region in the recA-amplicon region in the common ancestor of *C. fausta *and *C. pacifica *symbionts (Additional file 1, Figure S1: positions 1855-2123 for *C. fausta *symbiont and 1859-2125 for *C. pacifica *symbiont). Black triangle (6), Occurrence of large-sized deletions in recA- and mutY-amplicons in the common ancestor of clade I symbionts (Figure 2).

Before screening the genes for DNA recombination and repair, we analyzed the phylogenetic relationships of 12 symbionts of *Calyptogena *clams (Table 1). Because the resolution of the 16S rRNA tree was too low, we amplified and sequenced the 23S rRNA genes (Tables 2 and 3) from the symbionts and reconstructed trees using the concatenated 16S and 23S rRNA gene sequences (Figure 1). Two clades well supported with high bootstrap values and posterior probabilities were identified. Symbionts of *C. kawamurai*, *C. laubieri*, *C. kilmeri*, *C. soyoae *and *C. okutanii *(Vok) formed clade I, and those of *C. pacifica*, *C. fausta*, *C. nautilei*, *C. stearnsii*, *C. magnifica *(Rma), *C. fossajaponica *and *C. phaseoliformis *formed clade II (Figure 1). The bootstrap support for clade II [bootstrap values at the node in the maximum likelihood (ML) tree > 89, posterior probability = 1.00] was slightly less than that for clade I (bootstrap values = 100, posterior probability = 1.00). In clade II, the symbionts of *C. phaseoliformis *and *C. fossajaponica *formed a subclade with Rma, and the symbionts of *C. stearnsii*, *C. nautilei*, *C. fausta *and *C. pacifica *formed another subclade with a high bootstrap value (Figure 1). It is noteworthy that the branch length from the node of clade II (** in Figure 1) to Rma was longer than those of other branches in clade II, and that the branch length from the node of the radiation of the *Calyptogena *clam symbiont (* in Figure 1) to the node of clade I radiation (*** in Figure 1) was longer than that to clade II (** in Figure 1). In clade I, two subclades respectively containing symbionts of *C. kilmeri*, *C. soyoae *and *C. okutanii *(Vok) and those of *C. kawamurai *and *C. laubieri *were not robust (Figure 1).

**Table 1:** *Calyptogena *clams used in the present study

Host clam	Date of collection	Dive #	Depth (m)	Latitude	Longitude	Collection site
*Calyptogena phaseoliformis*	2006.6.3	6K#953	6264	40.10N	144.19E	Japan Trench
*Calyptogena fossajaponica*	2006.6.2	6K#952	6182	40.10N	144.17E	Japan Trench
*Calyptogena stearnsii*	1996.3.29	ROV Biv-0099-0103	659-683.5	36.77N	122.04W	Monterey Bay
*Calyptogena nautilei*	2005.6.18	6K#884	3306	32.58N	134.69E	Okinawa-Nankai Trough
*Calyptogena pacifica*	1996.3.29	ROV Biv-0105-0112	659-683.5	36.77N	122.04W	Monterey Bay
*Calyptogena fausta*	1996.6.10	2K#869	1490	34.91N	138.66E	Suruga Bay
*Calyptogena kawamurai*	2005.6.13	6K#881	608	34.08N	137.79E	Okinawa-Nankai Trough
*Calyptogena laubieri*	1997.8.23	10K#45	3761	33.65N	137.91E	Okinawa-Nankai Trough
*Calyptogena okutanii*	From database*					
*Calyptogena kilmeri*	1996.3.25	ROV Biv-0116-0121	900	36.73N	122.00W	Monterey Bay
*Calyptogena soyoae*	2000.7.1	HPD#13	1178	35.00N	139.23E	Sagami Bay off Hatsushima
*Calyptogena magnifica*	From database**					

HPD, *Hyper Dolphin*; 2K, *Shinkai 2000*; 6K, *Shinkai 6500*; ROV, remote operated vehicle; 10K, ROV *Kaiko*.

*Accession number AP009247.

**Accession number CP000488.

**Table 2:** Primers for PCR

Primer	Nucleotide sequence	Target gene	Coding amino acid sequence	Note
recA Fi	5'-GAYGCNGARCAYGCIYTNGAYCC-3'	*recA*	DAEHALD	ip
recA Ri	5'-ACNCCDATYTTCATNCKDATYTG-3'	*recA*	QIRMKIGV	ip
recA_F	5'-GATTGCATATCATTCATCTGATAACG-3'	*recA*	non	ep
recA_R	5'-AGTGGATTRGGATCAAGCATAGC-3'	*recA*	non	ep
mutY_F	5'-CCTATATGWACACCATCAAAGTTGCC-3'	*mutY*	non	ep
mutY_R	5'-AATACTTGGRGWAACGCCACTAATG-3'	*mutY*	non	ep
27F	5'-AGAGTTTGATCCTGGCTCAG-3'	16S	non	ip
1492R	5'-GGTTACCTTGTTACGACTT-3'	16S	non	ip
23S F1	5'-GGGAACTGAAACATCTAAGTACC-3'	23S	non	ip
23S R1	5'-CCCGCTTAGATGCTTTCAG-3'	23S	non	ip

ip, internal primer; ep, external primer.

16S, small subunit rRNA gene; 23S, large subunit rRNA gene.

**Table 3:** Accession numbers of symbiont DNA sequences determined in the present study or retrieved from databases

Host clam (Abbreviation of symbiont)	*recA *amplicon^#^	*mutY *amplicon^$^	16S rDNA	23S rDNA
*Bathymodiolus septemdierum *(Bsep S)	n.d.	n.d.	AB598130	AB598131
*Calyptogena phaseoliformis *(Cpha S)	AB586104	AB586113	AB479082*	AB598132
*C. fossajaponica *(Cfos S)	AB586105	AB586114	AB044744*	AB598133
*C. stearnsii *(Cste S)	AB642236	AB642237	AB642238	AB642239
*C. nautilei *(Cnau S)	AB586106	AB586115	AB479080*	AB598134
*C. pacifica *(Cpac S)	AB586107	AB586116	AF03572*	AB598135
*C. fausta *(Cfau S)	AB586108	AB586117	AB47908*	AB598137
*C. kawamurai *(Ckaw S)	AB586109	AB586118	AB479076*	AB598138
*C. laubieri *(Clau S)	AB586110	AB586119	AB073121*	AB598139
*C. okutanii *(Vok)	AP009247*	AP009247*	AP009247*	AP009247*
*C. kilmeri *(Ckil S)	AB586111	AB586120	AF035720*	AB598136
*C. soyoae *(Csoy S)	AB586112	AB586121	AB479077*	AB598140
*C. magnifica *(Rma)	CP000488*	CP000488*	CP000488*	CP000488*

#Gene region amplified with primers recA_F and recA_R.

$Gene region amplified with primers mutY_F and mutY_R.

n.d., Not determined in the present study.

*Not determined in the present study but retrieved from a database.

### DNA recombinase gene, *recA*, in *Calyptogena *clam symbionts

**Figure 2:**
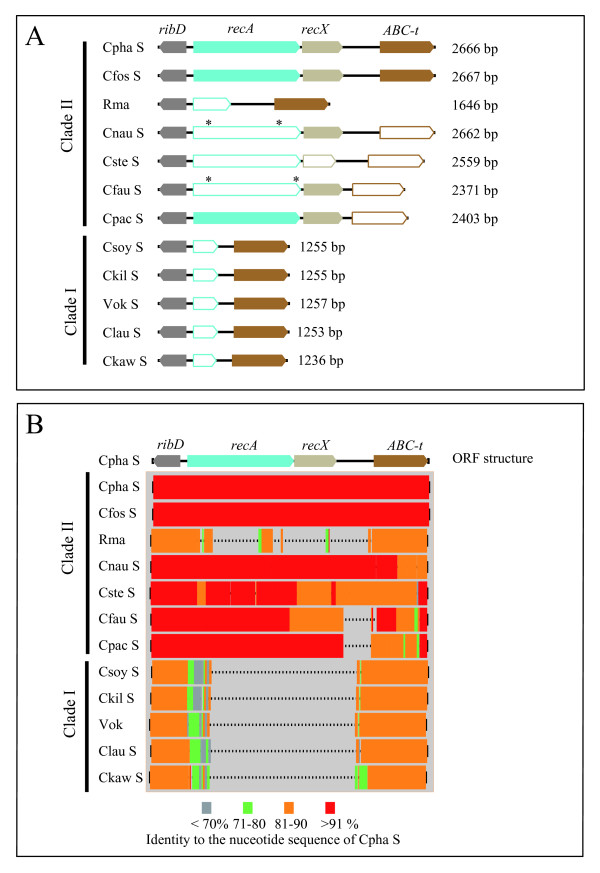
**Open reading frames and nucleotide sequence homologies in the recA-amplicons from *Calyptogena *clam symbionts**. A, Open reading frames (ORFs) of the recA-amplicons. Filled column, intact gene. Open columns, pseudogene or remnant gene. Gray column, part of the gene for RibD (bifunctional riboflavin biosynthesis protein). Blue column, *recA*. Light gray column, *recX*. Brown column, part of the gene for the ABC transporter ATP-binding protein (*ABC-t*). *, mutated stop codon. B, Aligned recA-amplicons showing their nucleotide sequence identities and deletion profiles with that of the *C. phaseoliformis *symbiont.

Using PCR with an internal primer set designed from the conserved sequences of *recAs *of several gamma-proteobacteria (Table 2), we obtained an amplicon from the genomic DNA of a *C. phaseoliformis *symbiont (data not shown). The sequence obtained was shown to be a part of *recA *by a BLASTX search in the DDBJ/GenBank/EMBL. A search for a residual *recA *in the Rma and Vok genomes using this sequence and the *Escherichia coli recA *sequence revealed that the Rma genome contains a DNA fragment similar to *recA *between Rmag_0799 (ABC transporter related, Figure 2A) and Rmag_0800 [diaminohydroxyphosphoribosylaminopyrimidine deaminase/5-amino-6-(5-phosphoribosylamino) uracil reductase, Figure 2A], but the Vok genome does not. The entire *recA *sequence and its flanking sequences from the *C. phaseoliformis *symbiont were amplified in PCR using the primers recA_F and recA_R (Table 2), which were designed from the franking conserved sequences of the *recA*-like sequence in Rma (Rmag 0799 and Rmag 0800) and their respective corresponding sequences in Vok [COSY_0725 (ABC transporter ATP-binding protein) and COSY_0726 (bifunctional riboflavin biosynthesis protein RibD)]. While Rmag_0800 and COSY_0726 are annotated with different gene names, they are homologous to each other.

In PCR using the primers recA_F and recA_R, amplicons with different lengths were obtained from 10 symbionts and sequenced (Table 3). These amplicons were designated as recA-amplicons. In open reading frame (ORF) analysis, we detected intact *recA *coding for 344-amino acid RecA in the symbiont genomes of *C. pacifica *and *C. fossajaponica *as well as *C. phaseoliformis *(Figure 2A). Their amino acid sequence identities to that of *E. coli *were 70.0%, 70.6% and 70.3%, respectively. However, Rma and the symbionts of *C. stearnsii*, *C. fausta *and *C. nautilei *were shown to have defective *recAs*. In Rma, highly degraded remnants of the *recA *gene were detected (Figure 2A). In the symbionts of *C. fausta *and *C. nautilei*, the *recA *was found to be degraded into a few apparent ORFs (Additional file 1 Figure S1). In both of the symbionts, the 52nd codon "GGT" was replaced with a stop codon "TAG." This was found to be caused by the same insertion of "CC" at the 151st-152nd base from the initiation codon of the original *recA *sequences (at the position between 483 and 484 in Additional file 1 Figure S1).

In the symbiont of *C. stearnsii*, the coding region of *recA *was found to be fragmented by many stop codons, which were caused by substitutions and a few base insertions, i.e., the substitution of "C" with "T" makes the stop "TGA" at the 343rd (10th base from the initiation site in *C. phaseoliformis recA*) and the insertion of "A" between the 427th and 428th base makes the stop "TAA" at 438-440 in Additional file 1 Figure S1 (Figures 2A and Additional file 1 Figure S1). No common insertion, deletion or substitutional mutation was detected between the symbiont of *C. stearnsii *and those of *C. nautilei *and *C. fausta*. In the symbionts of *C. pacifica*, *C. fossajaponica*, *C. phaseoliformis*, *C. fausta *and *C. nautilei*, an apparently intact ORF coding *recX *was found downstream of *recA *(Figure 2A). However, the symbiont of *C. stearnsii *was found to contain a pseudogene of *recX *(Figure 2A).

We performed multiple alignments of the recA-amplicons of the symbionts and analyzed the deletion profiles (Figure 2B). The longest amplicon was that of the symbiont of *C. phaseoliformis *or *C. fossajaponica*. They were thus thought to be the most similar to that of the common ancestor of clade I and II symbionts. In clade II symbionts, the nucleotide sequence of the recA-amplicon of the *C. fossajaponica *symbiont was the most similar to that of the *C. phaseoliformis *symbiont, or *vice versa*. However, the sequence identity of the *recA *coding region between symbionts of *C. phaseoliformis *and *C. fossajaponica *(90.1%) was lower than the identities of those of symbionts of *C. fausta*, *C. nautilei *and *C. pacifica *compared with that of the *C. phaseoliformis *symbiont (95.6%, 95.5% and 95.3%, respectively). To compare the sequences of recA-amplicons, we selected that of the *C. phaseoliformis *symbiont as a reference.

Compared with the recA-amplicon of the *C. phaseoliformis *symbiont, that of Rma had several deletions (Figure 2B). Deletions of almost the same size were found in symbionts of *C. fausta *(Figure 2B; from position 1857 to 2123 in Additional file 1 Figure S1) and of *C. pacifica *(Figure 2B; from position 1859 to 2125 in Additional file 1 Figure S1), while no such deletion was found in the symbionts of *C. stearnsii*, *C. nautilei *or *C. fossajaponica *(Figure 2B).

In clade I symbionts, *recA *was not recognized in their recA-amplicons. However, large deletions of a similar size were found in their respective recA-amplicons (Figure 2B). While some DNA fragments corresponding to the *N*-terminal RecA remained, *recA *was markedly disintegrated due to the large deletion (Figure 2B).

### DNA repair gene, *mutY*, in *Calyptogena *clam symbionts

**Figure 3:**
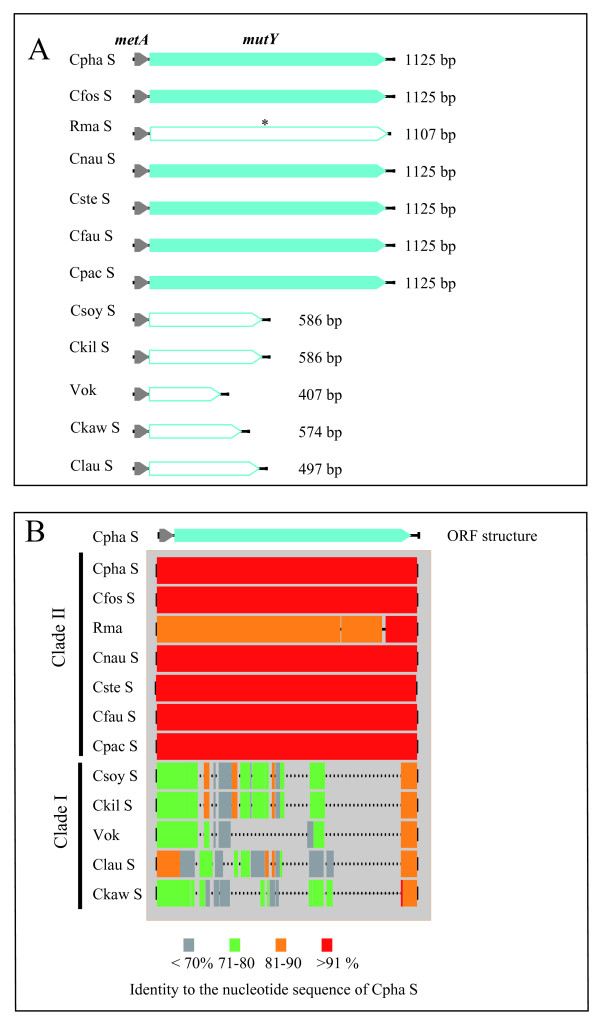
**Open reading frames and nucleotide sequence homologies in amplicons for the *mutY *region amplified from *Calyptogena *clam symbiont genomes**. A, ORFs in the mutY-amplicons. Filled pentagonal column, intact gene. Open pentagonal column, pseudogene or remnant of gene. Gray, part of *metA *(homoserine *O*-succinyltransferase). Blue, *mutY*. B, Aligned mutY-amplicons showing their nucleotide sequence identities and deletion profiles compared with that of the *C. phaseoliformis *symbiont. Dotted lines, deletions.

By performing a BLAST search of unannotated ORFs and pseudogenes in the Rma genome [16], we found that a translated amino acid sequence of Rmag_0017 (21165-22172) was homologous to MutY of other bacteria (48% identity to that of *Alteromonas macleodii *str. deep ecotype or 45% identity to that of *Thiomicrospira crunogena *XCL-2), but this gene was found to be a pseudogene due to the amber mutation "UGA" at codon 167. No such ORF was recognized in the Vok genome. A primer set, mutY_F and mutY_R, was designed to amplify the genomic region containing *mutY *(Table 2). The PCR products were designated as mutY-amplicons. An apparently intact *mutY *was found in each mutY-amplicon of the genomes of clade II symbionts except for Rma, i.e., symbionts of *C. phaseoliformis*, *C. fossajaponica*, *C. pacifica*, *C. stearnsii*, *C. fausta *and *C. nautilei *(Figure 3A), of which the gene product showed significant similarity to MutY of other bacteria. DNA sequence homology analysis with multiple alignment showed that the nucleotide sequences of mutY-amplicons from symbionts in clade II except for Rma showed greater similarity to that of the *C. phaseoliformis *symbiont (greater than 90%), while the identity with Rma was lower (80-90%) (Figure 3B). In addition, two small inframe deletions (3 bp and 15 bp; positions from 797 to 799 and from 974 to 988 in Additional file 2, Figure S2) were found in the nucleotide sequences of Rma *mutY*. On the other hand, the mutY-amplicons from clade I symbionts were much shorter than that of the *C. phaseoliformis *symbiont, showing that it was highly degraded by deletions and substitutions (Figure 3B).

Because MutY was expected to contribute GC bias to the genomes of the *Calyptogena *clam symbionts, we analyzed the G+C contents of their 16S and 23S rRNA genes (Table 4). The mean G+C content of the 16S rRNA genes in clade I symbionts and Rma, which lacked intact *mutY*, and that in clade II symbionts except for Rma were 49.01 ± 0.59 and 50.21 ± 0.29 (%: mean ± SD), respectively. This difference was significant in the *t*-test (p = 0.00119) (Table 4). Those of 23S rRNA genes in the symbionts lacking *mutY *(clade I symbionts and Rma) and in the symbionts with *mutY *(clade II symbionts except for Rma) were 48.25 ± 0.35 and 49.47 ± 0.36 (%: mean ± SD), respectively, and the difference was significant (p = 0.000143) in the *t*-test. The G+C contents of the 16S and 23S rRNA genes of Rma were 50.20% and 48.94%, respectively, which were intermediate between those of the symbionts with or without *mutY *(Table 4). This may indicate that *mutY *in Rma became a pseudogene relatively recently.

**Table 4:** Effect of mutY on the GC content of ribosomal RNA genes in *Calyptogena *clam symbionts

Host clam	Clade	mutY	16S rDNA		23S rDNA	
			
			Length (bp)	G+C%	Length (bp)	G+C%
*Calyptogena kilmeri *	I	-	1428	48.79	2519	48.14
*C. kawamurai *	I	-	1510	48.77	2519	47.94
*C. soyoae *	I	-	1508	48.77	2525	48.13
*C. laubieri *	I	-	1343	48.65	2519	48.25
*C*. *okutanii *	I	-	1468	48.87	2525	48.12
*C*. *magnifica*	II	-	1468	50.20	2536	48.94

Mean	I + Rma*			49.01#		48.25§
SD				0.59		0.35

Mean	I only			48.77$		48.12!
SD				0.08		0.11

*C. phaseoliformis*	II	+	1506	50.41	2530	49.81
*C. fossajaponica *	II	+	1467	50.64	2531	49.99
*C. stearnsii *	II	+	1467	49.90	2535	49.07
*C. nautilei *	II	+	1506	49.90	2528	49.36
*C. fausta*	II	+	1507	50.13	2534	49.31
*C. pacifica*	II	+	1428	50.27	2532	49.25

Mean	II except Rma*			50.21#$		49.47§!
SD				0.29		0.36

*Ca. Ruthia magnifica (symbiont of *C. magnifica*)

#Difference between G+C contents of 16S rRNA genes of clade I symbionts+Rma and of clade II symbionts except Rma was significant (p = 0.00119) in the *t*-test.

$Difference between G+C contents of 16S rRNA genes of clade I symbionts and of clade II symbionts except Rma (p = 0.00257) was significant in Welch's test.

§Differences between G+C contents of 23S rRNA genes of clade I symbionts+Rma and of clade II symbionts (p = 0.00014) was significant in the *t*-test.

!Difference between G+C contents of 23S rRNA genes of clade I symbionts and of clade II symbionts (p = 2.014e-5) was significant in the *t*-test.

## Discussion

Previously, we reported that *recA *was probably lost in the early stage of RGE in *Calyptogena *clam symbionts [8]. The present study showed that some of the extant clam symbionts still have intact *recA *(Figure 2). We hypothesized that in the early phase of RGE of the clam symbionts before the loss of *recA*, large-sized deletions occurred due to RecA-dependent recombination [8]. This type of deletion requires repeated sequences larger than 200 bp, which have been depleted from the genomes of Rma and Vok [8,19]. It is still not clear whether the genomes of the *Calyptogena *clam symbionts containing *recA *have large-sized (> 200 bp) repeated sequences. The presence of intact or of nearly intact *recA *and of *mutY *in clade II symbionts except for Rma suggests that the genomes of clade II symbionts are larger than those of clade I symbionts and that their RGE is in an earlier stage than in clade I symbionts. To resolve these questions, we must await their genome sequence analyses.

The coding region of *recA *was shown to be mostly deleted in Rma and clade I symbionts (Figure 2A and 2B). A similar large-sized deletion was found in each of the recA-amplicons of clade I symbionts (Figure 2B). This indicates that the shared part of their deletions occurred in the common ancestor of clade I symbionts after divergence from that of clade II symbionts [arrowhead (6) in Figure 1]. While both Rma and clade I symbionts lack *recA*, the phylogenetic tree strongly suggests that these losses occurred independently in both the ancestral Rma and the common ancestor of clade I symbionts (Figure 1).

Degradations of the ORFs for *recA *in Rma and in the symbionts of *C. stearnsii*, *C. fausta *and *C. nautilei *indicate that RGE in the extant clade II symbionts of *Calyptogena *clams is in the transitional stage of *recA *loss. The loss of *recA *may start with the degeneration of its ORF by point mutations or a few base insertion/deletion mutations like those in the symbionts of *C. fausta*, *C. nautilei *and *C. stearnsii *(Figures 2 and Additional file 2, Figure S1), then continue in the next stage with larger deletions, e.g., those in Rma and in clade I symbionts (Figures 2 and Additional file 2, Figure S1), generated by successive illegitimate recombinations or replication slippages without RecA [8,23]. This also suggests that the longer (> 200 bp) repeated sequences were depleted in the symbiont genome, and that as a result RecA was not able to function as a recombinase or a deletion generator in the genome before losing this gene.

A three-dimensional (3D) homology model of RecA reconstruction using the crystal structure of *E. coli *RecA [24] as a template showed that the 3D structure of RecA in the symbiont of *C. phaseoliformis *was similar to that of *E. coli *(Additional files 3 and 4, Figure S3). RecA consists of three domains: the *N*-terminal domain functions as a monomer-monomer interface; the central domain is responsible for ATP binding; and the *C*-terminal domain is responsible for dsDNA binding [24]. This indicates that RecA in the symbionts of *C. phaseoliformis*, *C. fossajaponica *and *C. pacifica *are functional, and that the truncated RecA in *C. fausta *and *C. nautilei *symbionts having only the *N*-terminal 68 amino acids is functionless (Additional files 3 and 4, Figure S3).

In the symbiont genomes of *C. fausta *and *C. nautilei*, the truncations of their *recAs *were respectively caused by the same two-base (CC) insertion mutations at the same position of the gene (Additional file 1, Figure S1). It is not clear whether the insertion occurred in the common ancestor of the symbionts of *C. fausta*, *C. nautilei *and *C. pacifica *[arrowhead (5) in Figure 1] and the inserted sequence was removed later in the symbiont of *C. pacifica*, or whether the insertions occurred independently in the two symbiont lineages of *C. nautilei *and *C. fausta *[arrowheads (3) and (4) in Figure 1]. If an insertion occurs randomly at any position of the genome, the identical two-base insertion would not likely have occurred independently at the same position of two different genomes at approximately the same time. This question should be addressed in future studies of their genomes.

Because no common insertion/deletion or substitutional mutation making a stop codon was detected among the symbionts of *C. stearnsii*, *C. fausta *and *C. nautilei*, the mutations in the *C. stearnsii *symbiont occurred independently in its lineage [arrowhead (2) in Figure 1].

The *recAs *of *C. fausta *and *C. nautilei *symbionts were shown to have additional insertions (Additional file 1, Figure S1). These insertions may have occurred after the loss of the function of the gene by the insertion of "CC" as a result of the relaxation of selective pressure. While RecA is known to be important for recombination and repair mutations, like double-strand breaks of DNA, intracellular symbionts tend to lose it [6]. The selective pressure to retain *recA *probably remained in the early evolutionary stages of the *Calyptogena *clam symbionts. However, after the loss of large-sized repeated sequences, the selective pressure for retaining *recA *may have decreased.

In clade II symbionts, the present data indicate that their *recAs *are currently deteriorating. This also supports the above hypothesis that the RGE stage due to *recA*-dependent deletion is probably ending in these extant genomes.

The DNA repair gene *mutY *was found in the genomes of clade II symbionts except for Rma (Figure 1). In Rma, *mutY *was found to be split into two ORFs (Figure 3A) by a substitution of the 501st G with A, making a new stop codon (Additional file 2, Figure S2). The phylogenetic tree indicates that this mutation occurred in the Rma lineage after divergence from the symbionts of *C. phaseoliformis *and *C. fossajaponica *[arrowhead (1) in Figure 1]. MutY has been shown to be composed of the *N*-terminal and *C*-terminal domains (Additional files 5 and 6, Figure S4) [25]. Substrate DNA binds to the cleft between these two domains [26]. While 3D homology modeling showed that MutY of *C. phaseoliformis*, *C. fossajaponica*, *C. fausta*, *C.nautilei *and *C. pacifica *symbionts seemed to have an intact 3D structure and to be functional (Additional files 5 and 6, Figure S4), the split gene products of the Rma *mutY *fragments are functionless (Additional files 5 and 6, Figure S4). The evidence that the gene encodes an almost intact amino acid sequence/architecture indicates that Rma lost the functionality of *mutY *relatively recently.

The G+C content of genomes generally tends to decrease in obligate intracellular symbionts with decreasing genome size [3]. MutY is known to repair A-G mismatches to C-G [20]. The loss of *mutY *in a genome is expected to decrease the G+C content [8,27]. However, many insect intracellular symbionts such as *Buchnera *spp. with genomes that have low G+C content still have *mutY *[8]. In addition, a recently found very small genome of the insect symbiont *Ca*. Hodgikinia cicadicola lacking *mutY *has a high G+C content [4]. These may contradict the above view and indicate that the loss of *mutY *does not significantly contribute to the decrease in the G+C content of the genome. However, in this study, the G+C content in the 16S and 23S rRNA gene sequences was significantly lower in the *Calyptogena *symbionts without *mutY *than that in the symbionts with *mutY *(Table 4). This supports the hypothesis that the loss of *mutY *contributes to the GC bias of the genome [20,27]. The G+C content of Rma was intermediate between the two symbiont clades. This agrees with the view that it lost functional *mutY *more recently than clade I symbionts during evolution. This result also coincides with the data showing that the G+C content of the Rma genome (34.0%) is higher than that of Vok (31.6%) [16,17]. Stewart et al. have recently reported that the G+C contents of 9 genes including 16S and 23S RNA genes of the symbionts in the *gigas/kilmeri *clade that corresponds to clade I in the present study were significantly lower than those of another clade that corresponds to clade II in the present study (Additional file 7, Figure S5) [28]. Although it was not clear whether the symbionts in the other clade reported by Stewart et al. [28] had *mutY *or not, the present results suggest that they do and thus the G+C contents of their genes are higher than those of the symbionts in the *gigas/kilmeri *clade.

It has recently been shown that mutational bias of GC→AT is a general trend in bacteria, and this trend may be counterbalanced by biased gene conversion and natural selection to maintain the G+C contents [29-31]. In intracellular symbionts, relaxation of natural selection, lower recombination frequency, small effective population size, codon usage, availability of nucleotides in the cytoplasmic pool and loss of DNA repair genes may contribute to lower G+C content [4,31,32]. In addition to the loss of *mutY*, any of these factors may have also contributed to a greater reduction of the G+C content in symbionts in clade I compared with those in clade II. However, this remains to be studied in future.

The present phylogenetic tree shows that both *mutY *and *recA *have been lost in Rma and in clade I symbionts (Figure 1). Were the losses in clade I symbionts and Rma accidental coincidences or related phenomena? The loss of *recA *may increase the mutation rate of the genome and hence increase the possibility of losing other genes such as *mutY*. It is also noteworthy that the branch length of Rma is longer than other branches in the clade II lineage, and the branch length from the node between clade I and II symbionts (* in Figure 1) to the node of clade I symbiont radiation (*** in Figure 1) is longer than the length to the node of clade II symbiont radiation (** in Figure 1). As a result, the loss of *recA *which occurred in Rma and clade I symbionts independently may have increased the mutation rate and elongated these branch lengths. This may also increase the probability of losing other genes including *mutY*.

Once genes lose their functions, their selective pressure must be relaxed and their mutation rates are expected to increase [33]. In the functionless *recAs *of *C. fausta *and *C. nautilei*, one additional mutation was found in each (Additional file 1, Figure S1). Two additional deletions were also found in the Rma *mutY *(Additional file 2, Figure S2). These may be the result of the decreased (relaxed) selective pressure after the losses of the functions of the genes.

While an evolutionary event like the loss of a gene for DNA repair or recombination may occur spontaneously in a certain lineage, it must greatly affect the later evolutionary fate of that lineage. We previously suggested that the loss of *recA *probably stabilized the genome architecture in *Calyptogena *clam symbionts [8,34]. The present data raise the possibility that the loss of *mutY *affected the G+C content of the genomes of the *Calyptogena *symbionts. The effect of losing genes for DNA recombination and repair on their RGE will be analyzed by sequencing the genomes of other *Calyptogena *clam symbionts, which is now in progress and will be published elsewhere.

## Conclusion

The apparently intact genes for DNA recombination and repair *recA *and *mutY *were found in some clade II symbionts, i.e., symbionts of *C. phaseoliformis*, *C. fossajaponica *and *C. pacifica*. Those of *C. stearnsii*, *C. nautilei *and *C. fausta *had intact *mutY *but their *recA *was found to be a pseudogene due to insertion/deletion and/or substitution mutations. These genes were disintegrated and lost in Rma and in clade I symbionts. Most of the *recA *coding region was lost in the common ancestor of clade I symbionts and in the Rma lineage as a result of deletions. In the symbionts of *C. stearnsii*, *C. fausta *and *C. nautilei*, *recA *became functionless due to small base insertions and substitutions. The *mutY *gene of Rma was disintegrated by a substitutional mutation. *mutY *was also lost through deletions in the common ancestor of clade I symbionts. The coinciding losses of both *recA *and *mutY *in Rma and clade I symbionts are thought to have occurred independently in the respective lineages.

The G+C contents of the symbionts with *mutY *were significantly higher than in those without *mutY*. This indicates that the loss of *mutY *probably decreased the G+C contents of the descendant symbiont genomes. This suggests that gene degradation, which occurs by chance in some lineages of symbionts, greatly affects the genomes of later descendants of the lineage.

## Methods

### Sample collection

*Calyptogena *clams were collected and stored at -80°C in a freezer until use (Table 1). *C. pacifica*, *C. stearnsii *and *C. kilmeri *were collected in Monterey Bay (Table 1) and were kind gifts to JAMSTEC from Dr. J. Barry of the Monterey Bay Aquarium Research Institute.

### DNA extraction

The gill tissue was dissected, washed with filtered (0.2-μm pore membrane filter; Millipore), sterilized artificial seawater to remove bacteria attached to the gill surface and chopped with scissors. DNA was extracted from approximately 10 mg of the tissue with a DNeasy Tissue Kit (Qiagen) according to the manufacturer's instructions. Although some bacteria attached to the surface of the gill tissue might have remained after washing, only the amplified products of the symbionts were detected in PCR for the 16S rRNA gene. This indicated that bacteria contaminating the surface of the gills were far less numerous than the symbionts.

### Primer design

Almost whole-length genes for the small subunit ribosomal RNA (16S rRNA) and the large subunit ribosomal RNA (23S rRNA) were amplified from 10 *Calyptogena *clam symbionts with specific primers (Table 2).

Based on the conserved RecA amino acid sequences of several gamma-proteobacteria, a set of internal consensus primers was designed for PCR amplification of *recA *in the *Calyptogena *clam symbionts (Table 2). In PCR, a DNA fragment amplified from *C. phaseoliformis *symbiont DNA was detected. A BLAST search showed that the DNA fragment obtained was a portion of *recA*. We then searched for *recA *or its remnant in the genomes of Rma and Vok with BLASTX using *recA *in *E. coli *str. 12, substr. MG 1655 (accession number = NC_000913, gene locus tag = b2699) as a reference and found a remnant gene sequence of *recA *in the Rma genome. A set of primers was designed from the conserved franking regions of the corresponding regions of the Rma and Vok genomes. The primers were designated as the external primers for *recA *(Table 2). Primers for PCR of *mutY *were designed based on the franking regions of the remnant sequences of *mutY *detected in the Rma genome and their corresponding sequences in Vok (Table 2).

### Amplification of the ribosomal RNA genes, *recA *and *mutY*, from *Calyptogena *clam symbionts

The genome regions containing *16S rRNA*, *23S rRNA*, *mutY*, *recA *or their corresponding regions were amplified by PCR with the primer sets shown in Table 2. According to the manufacturer's instructions, the reaction mixture contained 1 μl of template solution containing 100 ng of DNA, 5 μl of 10× ExTaq buffer (Takara), 4 μl of dNTP mix (Takara), 1 μl of the 10 pmol/μl forward primer solution, 1 μl of the 10 pmol/μl reverse primer solution, 0.25 μl of ExTaq polymerase solution (Takara) and 37.75 μl of pure water. The reaction mixture was initially incubated at 96°C for 2 min, then subjected to 35 cycles of the PCR protocol (96°C for 20 s, 55°C for 45 s and 72°C for 3 min) and finally to extension at 72°C for 10 min with a Takara TP600 Thermal cycler. The reaction mixture (2 μl) was applied to 1% agarose gel electrophoresis to check the amplicons. The gel was stained with 0.6% ethidium bromide solution to visualize the amplicon bands. The amplified DNA was purified with a Wizard SV Gel and a PCR Clean-Up System Kit (Promega) according to the manufacturer's instructions.

The nucleotide sequences of the amplified and purified DNAs were determined using a Big Dye Terminator v3.1 Cycle Sequencing Kit (Applied Biosystems) and an ABI PRIZM 3100 Genetic Analyzer (Applied Biosystems) according to the manufacturer's instructions. The sequences obtained were submitted to DDBJ-EMBL-GENBANK, and their accession numbers are listed in Table 3.

Phylogenetic relationships of *Calyptogena *clam symbionts were analyzed using the genes for 16S rRNA and 23S rRNA, some of which were retrieved from DDBJ-EMBL-GENBANK. The 16S and 23S rRNA gene sequences were concatenated and aligned using MAFFT 6 [34,35]. The alignment obtained was manually refined, and ambiguous nucleotide positions were excluded using Se-Al ver. 2.0all [36]. The aligned sequences (3128 bp) were analyzed using the neighbor-joining (NJ) and maximum composite likelihood methods [37] and maximum parsimony (MP) methods with MEGA 4 [38,39], as well as with the maximum likelihood (ML) method with PAUP* 4.0 [39]. The NJ tree was constructed with the maximum composite likelihood method distance [37]. MP analysis was performed with a heuristic search using close neighbor interchange (level = 1), a branch-swapping method with initial trees generated by random addition (10 replications); a complete deletion option was used to treat gaps/missing data. Modeltest ver 3.7 [40] was used to select the appropriate model of evolution for the ML analysis, with the Akaike information criterion. ML analysis was performed using the GTR+I+G model [41], and optimized parameter values were applied after the determination using Modeltest. The reliability of the tree topology was assessed by bootstrap resampling (number of pseudoreplicates: NJ and MP, 1000; ML, 100). They were also analyzed by the Bayesian method using MrBayes 3.1 [42]. The posterior probabilities were calculated to assess the reliability of the tree topology. For Bayesian analysis, we determined the optimal model of sequence evolution for each of the two genes (16S rRNA and 23S rRNA genes) using MrModeltest 2.2 [43]. The GTR+I+G model was selected for both the 16S rRNA and 23S rRNA genes. Bayesian analysis was performed with random starting trees and unlinked parameters and run for 5,000,000 generations, sampling the Markov chains at intervals of 100 generations. Four heated Markov chains (using default heating values) were used. The first 12,500 of the 50,000 resulting trees were discarded as "burn-in." To ensure that Markov chains were not trapped on local optima, Bayesian inferences were performed twice, beginning with different starting trees, and apparent stationary levels were compared for convergence [44].

To locate *recA *and *mutY *in the amplified DNA fragments, a search was performed for an ORF, and then a BLAST search against the NCBI protein database (nr) was performed. Multiple alignments for their nucleotide sequences were constructed using the Multi-LAGAN program [45].

## Authors' contributions

The experimental plan and story of this research were designed and organized by TdM. HK and SS performed the experiments and conducted resequencing to confirm the sequences. The phylogenetic analysis was carried out by TrM. DNA sequence data were analyzed by YT. TY reconstructed 3D homology models of the gene products. TK contributed to the discussion in this paper. All the data and analyzed results were examined carefully and discussed by all the authors. The final manuscript was read and approved by all the authors.

## Supplementary Material

Additional file 1 **Figure S1. Multiple sequence alignments of recA-amplicons from *Calyptogena *clam symbiont genomes**. The *recA*-containing genome region (recA-amplicon) was amplified with a primer set [recA_F (5'-GATTGCATATCATTCATCTGATAACG-3'), recA_R (5'-AGTGGATTRGGATCAAGCATAGC-3')] from 9 *Calyptogena *clam symbionts using the PCR and from 2 symbiont genomes [*Vesicomyosocius okutanii *(Vok: accession # = AP009247) and *Ruthia magnifica *(Rma: accession # = CP000488)] in *in silico *PCR. Abbreviations of symbionts are shown in Table 3. Gray background-colored horizontal sequence in Cpha S, part of *ribD*; blue background-colored horizontal sequence in Cpha S, *recA*; light gray background horizontal sequence in Cpha S, *recX*; brown background horizontal sequence of Cpha S, part of *ABC-t *(ABC transporter ATP-binding protein gene). *Identical nucleotide in the aligned sequences. Gray background vertical column, gap in Cpha S sequence. Red letters, in-frame start codon of *recA *or mutated *recA *ORFs. Blue letters, in-frame stop codon. Click here for file

Additional file 2 **Figure S2. Multiple sequence alignment of mutY-amplicons from *Calyptogena *clam symbiont genomes**. The *mutY*-containing genome region (mutY-amplicon) was amplified with a primer set [mutY_F and mutY_R (Table 2)] from 9 *Calyptogena *clam symbionts in PCR and from 2 symbiont genomes [*Vesicomyosocius okutanii *(Vok: accession # = AP009247) and *Ruthia magnifica *(Rma: accession # = CP000488)] in *in silico *PCR. Abbreviations of symbionts are shown in Table 3. Gray background horizontal sequence in Cpha S, part of *metA *(homoserine *O*-succinyltransferase); blue background sequence in Cpha S, *mutY*. *Identical nucleotide in the aligned sequences. Gray vertical column, gap in Cpha S sequence. Red letters, in-frame start codon of the original or mutated *mutY *ORFs. Blue letters, in-frame stop codon. Click here for file

Additional file 3 **Figure S3. Part A. 3D homology models reconstructed for RecA of the *Calyptogena phaseoliformis *symbiont**. Homology modeling using the Swiss-Model Workspace (http://swissmodel.expasy.org/) was based on the 3D structure of *Escherichia coli *RecA (PDB accession number 1U94: [24]) as a template. A, Alignment of amino acid sequences of *Calyptogena *clam symbiont RecA and *E. coli*. RecA. Sequences were aligned with ClustalW. a, RecA sequence from Lys-6 to Pro-331 in *E. coli*; b, secondary structure of *E. coli *RecA (1U94). Red rectangles, α-helices; blue arrows, β-strands. c, RecA sequence from Lys-5 to Thr-329 in the symbiont of *C. phaseoliformis*. d, RecA sequence of *N*-terminal ORF in the symbiont of *C. fausta*. e, RecA sequence of *C*-terminal ORF in the symbiont of *C. fausta*. Click here for file

Additional file 4 **Figure S3. Parts B-E. 3D homology models reconstructed for RecA of the *Calyptogena phaseoliformis *symbiont**. Homology modeling using the Swiss-Model Workspace (http://swissmodel.expasy.org/) was based on the 3D structure of *Escherichia coli *RecA (PDB accession number 1U94: [24]) as a template. B, Homology model reconstructed for *C. phaeoliformis *symbiont RecA. C, Homology model reconstructed for *N*-terminal and *C*-terminal amino acid peptides of RecA in *C. fausta *symbiont. *N*-terminal and *C*-terminal peptides are shown in violet and light blue, respectively. D, Crystal structure of *E. coli *RecA (accession # = 1U94); α-helices and β-sheets are indicated in red-green, and light blue, respectively. E, Merged 3D structures of RecAs of *E. coli *(D) and of *C. phaseoliformis *symbionts (B) showing that their 3D structures are nearly the same. This suggests that the *C. phaseoliformis *symbiont RecA is intact and functional. Click here for file

Additional file 5 **Figure S4. Part A. 3D homology models reconstructed for MutY of the *C. phaseoliformis *symbiont**. The model was reconstructed using the Swiss-Model Workspace (http://swissmodel.expasy.org/) based on the crystal structure of *Geobacillus stearothermophilus *MutY [26] (PDB accession # = 3FSP) as a template. A, Alignment of MutY amino acid sequences of *Calyptogena *symbionts and *G. stearothermophilus*. Sequences were aligned using ClustalW. a, MutY sequence from Phe-8 to Ser-360 in *G. stearothermophilus*. b, Secondary structure of *G. stearothermophilus *MutY (3FSP). Red rectangles, α-helices; blue arrows, β-strands. c, MutY sequence from Val-1 to Asp-341 in the symbiont of *C. phaseoliformis*. d, MutY sequence of *N*-terminal ORF in *Ruthia magnifica*. e, MutY sequence of *C*-terminal ORF in *R. magnifica*. Click here for file

Additional file 6 **Figure S4. Parts B-E. 3D homology models reconstructed for MutY of the *C. phaseoliformis *symbiont**. B, Homology model reconstructed for *C. phaseoliformis *symbiont MutY. C, Homology model reconstructed for *N*-terminal (violet) and *C*-terminal (light blue) amino acid peptides of MutY in *R. magnifica*. D, Crystal structure of *G. stearothermophilus *MutY (accession # = 3FSP). α-Helices and β-sheets are indicated as red-green and light blue, respectively. E, Merged 3D structures of MutY of *G. stearothermophilus *(D) and *C. phaseoliformis *symbionts (B). Click here for file

Additional file 7 **Figure S5. Phylogenetic tree of the *Calyptogena *clam symbionts including those of reported in Stewart et al. 2009 **[28]. 16S and 23S rRNA gene sequences of the symbionts reported in the present study and of those reported in Stewart et al. (2009) [28] were concatenated and used for phylogenetic tree reconstruction. Topology of the tree constructed using the maximum likelihood method is shown with bootstrap values (> 50%) obtained from the neighbor joining and maximum likelihood methods at each node. Accession numbers of the sequences are shown in the tree. Names and abbreviations of the symbionts are the same as those in Table 3 of the present study or those in [28]. Abbreviations for generic names: *C*., Calyptogena; *E*., Ectenagena; *V*., Vesicomya. *Symbionts reported in Stewart et al. [28]; **symbionts reported in both Stewart et al. [28] and the present study. Click here for file
