# Supplementary material for: Loss of genes for DNA recombination and repair in the reductive genome evolution of thioautotrophic symbionts of Calyptogena clams
Source: BMC Evol Biol. 2011 Oct 3;11:285. doi: 10.1186/1471-2148-11-285 (PMC3202245; doi:10.1186/1471-2148-11-285)
Supplement: Additional file 1 — Figure S1. Multiple sequence alignments of recA-amplicons from Calyptogena clam symbiont genomes. The recA-containing genome region (recA-amplicon) was amplified with a primer set [recA_F (5'-GATTGCATATCATTCATCTGATAACG-3'), recA_R (5'-AGTGGATTRGGATCAAGCATAGC-3')] from 9 Calyptogena clam symbionts using the PCR and from 2 symbiont genomes [Vesicomyosocius okutanii (Vok: accession # = AP009247) and Ruthia magnifica (Rma: accession # = CP000488)] in in silico PCR. Abbreviations of symbionts are shown in Table 3. Gray background-colored horizontal sequence in Cpha S, part of ribD; blue background-colored horizontal sequence in Cpha S, recA; light gray background horizontal sequence in Cpha S, recX; brown background horizontal sequence of Cpha S, part of ABC-t (ABC transporter ATP-binding protein gene). *Identical nucleotide in the aligned sequences. Gray background vertical column, gap in Cpha S sequence. Red letters, in-frame start codon of recA or mutated recA ORFs. Blue letters, in-frame stop codon. [file 1471-2148-11-285-S1.PDF]

```
position                                                                                                   1                   1
                    1         2         3         4         5         6         7         8         9         0         1         2
           123456789012345678901234567890123456789012345678901234567890123456789012345678901234567890123456789012345678901234567890

Cpha S     AGACGGGGTTTTACCTTGGTGTGAGCAAGGTTCTAAGGTGATGTAAAACGTGGCGTCCTGAGCTTGATGGTTAATTTGTTGTAAGGCGTTAATTTCCCCGTGCGCTTTGCCAAAGGTTTG
Cfau S     AGACGGAGTTTTGCCTTGATGCGAACAAGGTTCTAAGGTGACGTAAAACGTGGCGTCCTGAGCTTGATGATTAATTTGTTGTAAGGCGTTAATTTCTCCGTGTGCTTTGCCAAAGGTTTG
Cnau S     AGACGGAGTTTTACCTTGGTGTGAACAAGGCTCTAAGGTGACGTAAAACGTGGCGTCCTGAGCTTGATAGTTAATTTGTTGTAAGGCGTTAATTTCCCCGTGTGCTTTACCAAAGGTTTG
Cste S     AGATGGAGTTTTGCCTTGGTACGAACAAGGTTCTAAGGTGACGTAAAACGTGGCGTCCTGAGCTTGATGGTTAATTTGTTGTAATGCGTTAATTTCCCCGTGTGCTTTGCCAAAGGTTTG
Cpac S     AGACGGAGTTTTGCCTTGGTGCGAACAAGGTTCTAGAGTGACGTAAAACGTGGCGCCCTGAGCTTGATGGTTAATTTGTTGTAAGGCGTTAATTTCTCCGTGTGCTTTGCCAAAGGTTTG
Cfos S     AGACGGGGTTTTTCCTTGGTGTGAACAAGGCTCTAGAGTGACGTAAAATGTAGCATTCTGAGCTTGGTGGTTAATTTGTTGTAAGGCGTTAATTTCCCCATGCGCTTTGCCAAAGGTTTG
Rma S      AGATGGAGTTTTTCCTTGATGTGAACAAGGTTCTAAGGTGACGTAAAATGTAGCACCCTGAGCTTGGTGGTTAATTTGTTGTAAGGCGTTAATTTCCCCGTGCGCTTTACCAAAGGTTTG
Ckaw S     AGATGGTGTTTTTCCTTGATGTGAACAAGGTTCTAGAGTGACGTAGAGTGTAGCACCGTGAGCTTTATAGTTAATTTGTTGTAAGGCATTAATTTCTCCGTGTGCTTGGCCAAAATTTTG
Clau S     AGATGGTGTTTTTCCTTGATGTGAACAAGGTTCTAGAGTGACGTAGAGTGTAGCGCCATGAGCTTTATAGTTAATTTGTTGTAAGGCATTAATTTCTCCGTGTGCTTGGCCAAAACTTTG
Ckil S     AGATGGTGTTTTTCCTTGATGTGAACAAGGTTCTAGAGTGACGTAGAGTGTAGCGCCATGAGCTTTATAGTTAATTTGTTGTAAGGCATTAATTTCCCCGTGCGCTTGGCCAAAACTTTG
Csoy S     AGATGGTGTTTTTCCTTGATGTGAACAAGGTTCTAGAGTGACGTAGAGTGTAGCGCCATGAGCTTTATAGTTAATTTGTTGTAAGGCATTAATTTCCCCGTGCGCTTGGCCAAAACTTTG
Vok  S     AGACGGTGTTTTTCCTTGATGTGAACAAGGTTCTAGAGTGACGTAGAGTGTAGCGCCATGAGCTTTATAGTTAATTTGTTGTAAGGCATTAATTTCTCCGTGTGCTTGGCCAAAACTTTC
           *** ** ***** ***** *  ** ***** ****  **** *** *  ** **    *******  *  ************* ** ******** ** ** ****  *****  ***

position   1                                                                      2                                       2
           2        3         4         5         6         7         8         9         0         1         2         3         4
           123456789012345678901234567890123456789012345678901234567890123456789012345678901234567890123456789012345678901234567890

Cpha S     GTGATAGCCTTTGGCAATAATTTTTGCATGTTTTACAATCACACAACCGACCATAGGATTAGACTTTACACCGTATATTCCTTGACTGGCAAGTTTGAGTGCGATTGCCATATTTTGAGT
Cfau S     GTGATAGCCTTTGGCAATAATTTTTGCATGTTTTACAATCACACAACCGACCATAGGATTAGACTTTACACTGTATATGCCTTGACTGGCAAGTTTGAGTGCGATTGCCATGTTTTGAGT
Cnau S     GTGATAGCCTTTGGCAATAATTTTTGCATGTTTTACAATCACACAACCGACCATAGGATTAGACTTTACACCGTATATTCCTTGACTGGCAAGTTTGAGTGCGATTGTCATGTTTTGAGT
Cste S     GTGATAGCCTTTGGCAATAATTTTTGCATGTTTTACAATCACACAACCGACCATAGGATTAGACTTTACTTCGTATATTCCTTGACTGGCAAGTTTGAGTGCGATTGCCATGTTTTGAGT
Cpac S     GTGGTAGCCTTTGGCAATAATTTTTGCATGTTTTACAATCACACAACCGACCATAGGATTAGACTTTACACCGTATATCCCTTGACTGGCAAGTTTGAGCGCGATTGCCATGTTTTGAGT
Cfos S     GTGATAGCCTTTGGCAATAATTTTTGTATGTTTTACAATTACACAACCAACCATAGGATTGGACTTTACACCGTATTTTCCTTGGCTGGCAAGTTTGAGTGCAATCGTCATGTTTTGAGT
Rma S      GTGGTAGCCTTTGGCAATAATTTTTGTATGTTTTACAATCACACAACCGACCATAGGATTGGACTTTACACCGTATTTTCCTTGGCTGGCAAGTTTGAGAGCAATCACCATATTTTGAGT
Ckaw S     GTGATATCCTTTGGCAATAATCTTGGAATGTTTAACAATCACACAACCTACCATAGGATTAGACTTTACACCATACCTTCCTTGCAAGGCAAGTTTGAGTGCAATTGTCATATTTTTGGT
Clau S     GTGATATCCTTTGGCAATAATCTTGGAATGTTTAACAATCACACAACCTACCATAGGATTAGATTTTACACCGTATCTTCCTTGTAAGGCGAGCTTAAGTGCGATTGTCATGTTTTGGGT
Ckil S     GTGATATCCTTTGGCAATAATCTTGGAATGTTTAACAATCACACAACCTACCATAGGATTAGACTTTACACCGTATCTTCCTTGTAAGGCAAGTTTGAGTGCTATTGTCATGTTTTGGGT
Csoy S     GTGATATCCTTTGGCAATAATCTTGGAATGTTTAACAATCACACAACCTACCATAGGATTAGACTTTACACCGTATCTTCCTTGTAAGGCAAGTTTGAGTGCTATTGTCATGTTTTGGGT
Vok  S     GTGATATCCTTTGGCAATAATCTTGGAATGTTTAACAATCACACAACCTACCATAGGGTTAGACTTTACACCGTACCTTCCTTGTAAGGCAAGTTTAATTGCAATTGTCATGTTTTGGGT
           *** ** ************** ** * ****** ***** ******** ******** ** ** *****    **  * *****    *** ** ** *  ** **    *** ****  **

position   2                                                           3                                                      3
           4        5         6         7         8          9         0          1         2         3         4         5       5
           12345678901234567890123456789012345678901-2345678901234567890 12-34567890123456789012345678901234567890123456789012345678
in recA                                                                                                    1                   25
Cpha S     GTCATTTATTGAAAAAGTTGCCGGCATATTTCAGATATAAT-TGTGAAAATATTTTTCATAAG-AAATAACAACATTTTACCTAGGAGAAACAACGTGGATGAACAGAAAAAACAAGCCT
Cfau S     GTCATTTTTTGAAAAAGTTGCCGGCATATTTCAGATATAAT-TGTGAGAATATTTTGCATAAA-CATTGATAATATTTTACCTAGGAGAAACAACATGGATGAACAAAAAAAACAAGCCT
Cnau S     GTCATTTATTGAAAAAGTTGCCGGCATATTTCAGATATAAT-TGTGAAAATATTTTTCATAAA-AAATAACGATATTTTACCTAGGAGAAACAACATGGATGAACAGAAAAAACAAGCCT
Cste S     GTCATTTATTGAAAAAGTTGTCGGCATATTTCAGATATAAT-TGTGAAAATTTCTTGCATAAG-AAATAACAACATTTTACCTAGGAGAAACAACATGGATGAATAGAAAAAACAAGTCT
Cpac S     GTCATTTATTGAAAAAGTTGCCGGCATATTTCAGATATAAT-TGTGAGAATATTTTGCATAAG-AATTAACAATATTTTACCTAGGAGAAACAACATGAATGAACAGAAAGAACAAGCCT
Cfos S     ATCATTTATTGAAAAAGTTGCCGGCATATTTCAGATATAATCTGTGAAAATATTTTGCATAAG-AATTGATAACATTTTACCTAGGAGAAACAACGTGGATGAACAGAAAAAACAAGCCC
Rma S      GTCATTTTTTGAAAAAGTTGCTGACATATTTCAGATACAAT-TATGAAAACAGTTTACATAAG-AATTAACAACATTTTACCTAGAAGAAACAATGTAGATGAATAGAAAAAATAAGTCC
Ckaw S     TTCATTTTTTGAAAAAGTTGTTAGCATATTTCAGATATAAT-TGTGAAAATATTTTGCATAAACAATTAATATTATTTTACCTAGGAAAAATAACATGGATGAACAGAAAAAATAAGCCC
Clau S     TTCATTTTTTGAAAAAGTTGTTAGCATATTTCAGATATAAT-TGTGAAAATATTTAGCATAAACAATTAATAGTATTTTACCTAGGAAAAACAACATGGATGAACAGAAAAAATAAGTCC
Ckil S     TTCATTTTTTGAAAAAGTTGTTAGCATATTTCAGATATAAT-TGTGAAAATATTTTACATAAACAATTAATAATATTTTACCTAGGAAAAAGAACATGGATGAACAG-AAAAATAAGTCC
Csoy S     TTCATTTTTTGAAAAAGTTGTTAGCATATTTCAGATATAAT-TGTGAAAATATTTTACATAAACAATTAATAATATTTTACCTAGGAAAAAGAACATGGATGAACAG-AAAAATAAGTCC
Vok  S     TTCATTTTTTGAAAAAGTTGTTAGCATATTTCAGATATAAT-TGTGAAAATATTTTGCATAAACAATTAATAATATTTTACCTAGGAAAAAGAACATAGATGAACAG-AAAAATAAGTCC
            ****** ************    ************* *** * *** **    *  *****   * * *    *********** * *** **  *  ***** *  ** ** *** *

           3                                          4                                                                       4
position   56           7         8         9         0           1         2          3         4         5         6        7  7
           901--23456789012345678901234567890123456789012345--67890123456789012345678901234567-890123456789012345678901234567890123
in recA                                                                                    95                                  140
Cpha S     TAA--AAGCAACCCTTGCACAGATTGATAAACAGTTTGGCAAGGGTTCA--GTTATGTTTTTGGGCGATGACC-AGGCTCAAAGTGATATTGAGGCAGTGTTTACAGGTAGTTTAAGCCT
Cfau S     TAA--AAGCAGCTCTTGCACAGATTGATAAACAATTTGGCAAGGGTTCA--GTTATGTTTTTGGGCGATGACC-AAGTTCAAAGTAATATTGAGGCAGTTTCTACAGGCAGTTTAGGCTT
Cnau S     TAA--AAGCAGCCCTTGCACAGATTGATAAACAATTTGGCAAGGGTTCA--GTTATGTTTTTGGGCGATGACC-AAGCTCAAAGTAATATTGAGGTAGTTTCTACAGGCAGTTTAAGCTT
Cste S     TAA--AAGCAGCTCTTGTACAGATTGATAAACAATTTGGCAAGGGTTTA--GTTATGTTTTTGGGCAATGACCAAAGCTCAAAGTAATATTGAGGTAGTTGCTACAGGCAGTTTAAGATT
Cpac S     TAA--AAGCAGCCCTTGCACAAATTGATAAACAATTTGGCAAGGGTTCA--GTTATGTTTTTGGGCGATGACC-AAGCCCAAAGCAATATTGAGGCAGTTTCTACAGGCAGTTTAAGCTT
Cfos S     TAA--AAGCAGCTCTTGCACAGATTGATAAGCAATTTGGCAAAGGCTCA--GTTATATTTTTAGGCGACGATC-AAGCTCAAAGTGATATTGAGGTGGTTTCTACAGGTAGTTTAAGTCT
Rma S      TAA--TGACAGCTTTTACGCAGATTGATAAGCAATTTGACAAAAACTCA--GTTATGTTTTTTGGCGAGGATT-AAGCTCAAAGCGATA-TGAGGTAGTTTCTACAGGCAGTTTAAG---
Ckaw S     TAT--AAGTAGCCC--GTACAGATTGATAAATGTTTGG---------------TATATTCTGAGGCGATTATT-AGACTTAAAGTGATATTAAAGCAGTTTTTATAGGAAATGTAAGTTT
Clau S     TAT--AAGTAGTCC--GTAAAGATTGATAAATAATTTGATAAAGGTTTA--GTTATATTTTGAGGATATTATT-AGACTTAAAGTGATATTAAAGCAGTTTTTATAGGAAATGTAAATTT
Ckil S     TATA-AGTAAGTCC--GTACAGATTGATAAATAATTTTATAAAGGTTTGAGATTATATTTTGAGGTGATTATT-AGACTAAAAGTGATATTAAAGTAGTTTTTATAGGAAATGTAAGTTT
Csoy S     TATA-AGTAAGTCC--GTACAGATTGATAAATAATTTTATAAAGGTTTGAGATTATATTTTGAGGTGATTATT-AGACTAAAAGTGATATTAAAGTAGTTTTTATAGGAAATGTAAGTTT
Vok  S     TATATAAGTAGTTC--GTACAGATTGATAAATAATTTTATAAAGGTTT--GGTTATATTTTGAGGTAATTATT-AGACTAACAGTGATATTAAAGCAGTTTTTATAGGAAATGTAAGTTT
           **       *          * ********    **                   *** ** *  **  *  *   *     * **  *** * * *  **   ** *** * * **

position   4                            5                                                                                     5
           7     8           9         0         1         2          3         4         5            6         7         8  8
           4567890123--45678901234567890123456789012345678901234567 8-9012345678901234567890123456 7---890123456789012345678901234567
in recA                 151                                                                                                   254
Cpha S     AGATATTGCC--CTAGGTATTGGTGGTTTACCTAGGGGGAGAGTGATCGAAATTTAT-GGTCCTGAGTCATCAGGTAAAACAACCTT---GACATTGCACGTGATTGCTGAGGTACAAAA
Cfau S     AGATATTGCCCCCTAGGTATTGGTGGTTTACCTAAGGGGAGAGTGATCGAAATTTAT-GGTCCTGAGTCATCAGGCAAAACAACGCT---AACATTGCACGTGATTGCTGAGGTACAAAA
Cnau S     AGATATTGCCCCCTAGGTGTTGGTGGTTTACCTAGGGGGAGAGTGATCGAAATTTAT-GGTCCTGAGTCACCAGGTAAAACAACGCT---GACATTGCACGTGATTGCTGAGGTACAAAA
Cste S     GAACATTGCC--CTAGGTATTGGTGGTTTACCTA-GGGGAGAGTGATTTAAATTTAT-GGTTCTGAGTCATCAGGCAAAACAACGCT---GACATTGCACGTGATTGCTGAGATACAAAA
Cpac S     AGACATTGCC--CTAGGTATTGGTGGTTTGCCTAGGGGGAGAGTGATCGAAATTTAT-GGTCCTGAGTCATCAGGCAAAACAACGCT---GACATTGCACGTGATTGCTGAGGTACAAAA
Cfos S     AGATATTGCC--TTAGGTATTGGTGGCTTGCCTAGGGGGCGAGTGGTTGAAATTTAT-GGTCCTGAGTCATCAGGCAAAACAACGCT---GACATTGCACGTGATTGCCGAGGTGCAAAA
Rma S      -------------------ATGGCGATCTATCTAG-----GAGTAACCTAAATTTAT-GGTCCTGAGTTATCAGGTAAAATAACACT---GATATTGTAGATGATTGCTGAGATGAAAAA
Ckaw S     AATTTTAA-----TTTACATTAATGTCACCTAAGGAG-TAGAGTAATTGAAATTTACAAACCCTTATTTATTAGGTAAA--AACCCT---AATATTGCATGTAATTG------------
Clau S     AATTTTAA-----CTTACATTAATATCACCCCAATGGGTAGAGTGATTGAAATTTATAAACCCTTATTTATCAGGTAAA--AGTCCT---AACATTACATCTGATTG------------
Ckil S     AATTTTAA-----CTTACATTAATATCACCTAAGAGGTTAGAGTAATTGAAATTTATAAACCCTTATTTGTCAGGTAAA--AACCCT---AACATTACATGTGATTG------------
Csoy S     AATTTTAA-----CTTACATTAATATCACCTAAGAGGTTAGAGTAATTGAAATTTATAAACCCTTATTTGTCAGGTAAA--AACCCT---AACATTACATGTGATTG------------
Vok  S     AATTTTAA-----CTTACATTAATGTCACCTAAGGAGTTAGAGTAATTGAAATTTATAAACCCTTATTTATTAGGTAAA--AACCCTAACAACATTACATGTGATTG------------
                                *                   ****     *******      ** * *    *** ***  *    *    * ***  *  * ****

position   5           6                                                                                           7      7
           8 9         0         1         2         3         4         5         6         7         8         9         0      0
           890123456789012345678901234567890123456789012345678901234567890123456789012345678901234567890123456789012345678901234567
in recA                                                                                                                       374
Cpha S     ACTAGGTGGTACAGCGGCCTTTATTGATGCTGAGCATGCGCTTGATCCTAGATACGCTAAACGTTTGGGTGTTAACACGGATGATTTGTTAATTTCACAACCCGATACAGGTGAGCAAGC
Cfau S     ACTAGGTGGTACAGCGGCTTTTATTGATGCTGAGCATGCGCTTGATCCTAGATACGCTAAACGTTTGGGTGTTAATACGGATGATTTGTTAATTTCACAACCCGATACAGGTGAGCAAGC
Cnau S     ACTAGATGGTACAGCGGCCTTTATTGATGCTGAGCATGCACTTGATCCTAGATACGCTAAACGTTTGGGTGTTAATACGGATGATTTGTTAATTTCACAATCTGATATAGGTGAGCAAGC
Cste S     ACTAGGCGGTACAGCGGCCTTTATTGATGCTGAGCATGCGCTTGATCCTAGATACGCTAAACGTTTGGGTGTTAATACGGATGATTTATTAATTTCACAACCCGATACAGGTGAGCAAGC
Cpac S     ACTAGGTGGTACAGCGGCCTTTATTGATGCTGAACATGCGCTTGATCCTAGATACGCTAAACGTTTGGATGTTAATACGGATGATTTGTTAATTTCACAACCTGATACAGGTGAGCAGGC
Cfos S     ACTAGGTGGCATAGCAGCCTTTATTGATGCTGAACATGCGCTTGACCCTAAATATGCTAAATGTTTGGGCGTTAATACGGACGATTTATTAATTTCACAGCCTGATACTGGTGAGCAAGC
Rma S      CC----------------------------------------------------------------------------------------------------------------------
Ckaw S     ------------------------------------------------------------------------------------------------------------------------
Clau S     ------------------------------------------------------------------------------------------------------------------------
Ckil S     ------------------------------------------------------------------------------------------------------------------------
Csoy S     ------------------------------------------------------------------------------------------------------------------------
Vok  S     ------------------------------------------------------------------------------------------------------------------------
```

```
position  7                                                                                               8                      8
          0 1         2         3             4         5         6         7         8         9         0         1         2  2
          8901234567890123456789012345----67890123456789012345678901234567890123456789012345678901234567890123456789012345678901234567890123
in recA                                   403                                                                                     490
Cpha S    ATTAGAGATTACGGATATGTTAGTGCGC----TCAGGTAGCGTGGATATTGTGGTGATTGACTCTGTTGCAGCACTAACACCCAAGGCAGAAATAGAAGGTGAAATGGGCGCTTCACATA
Cfau S    ATTAGAGATTACGGATATGTTAGTACGC----TCAGGTAGCGTGGATATTGTGGTGATTGACTCTGTTGCAGCACTCGCACCCAAGGCAGAAATAGAGGGTGAAATGGGTGCTTCACATA
Cnau S    ATTAGAGATTACGGATGTGTTAGTGCGC----TCAGGTAGTGTGGATATTATGGTGATTGACTCTGTTGCAGCACTTGCACCCAAGGCAGAAATAGAGGGTGAAATGGGTGCTTCACATA
Cste S    ATTAGAGATTATGGATATGTTAGTGTGCGCTTTCAGGTAGCGTGGATATTGTGGTGATTAACTCTGTTGCAGCACTCGCACCCAAGGTAGAAATAAAAGGTGAAATGGGTGCTTCACATA
Cpac S    ATTAGAGATTACGGATATGTTAGTGCGC----TCAGGTGGCGTGGATATTGTGGTGATTGACTCTGTTGCAGCACTCGCACCCAAGGCAGAAATAGAGGGTGAAATGGGCGCTTCACATA
Cfos S    ATTGGAAATTACAGATATGTTGGTGCGT----TCAGGTAGTGTGGATATTGTAGTGATTGACTCTGTTGCGGCATTAACGCCCAAGGCAGAAATAGAGGGTGAAATGGGCGCTTCACATA
Rma S     ------------------------------------------------------------------------------------------------------------------------
Ckaw S    ------------------------------------------------------------------------------------------------------------------------
Clau S    ------------------------------------------------------------------------------------------------------------------------
Ckil S    ------------------------------------------------------------------------------------------------------------------------
Csoy S    ------------------------------------------------------------------------------------------------------------------------
Vok  S    ------------------------------------------------------------------------------------------------------------------------

position  8                                                                           9                                          9
          2     3         4         5         6         7         8         9         0         1         2         3         4  4
          456789012345678901234567890123456789012345678901234567890123456789012345678901234567890123456789012345678901234567890123
in recA   491                                                                                                                     610
Cpha S    TGGGACTTCAAGCCCGACTTATGTCTCAAGCACTTAGAAAACTCACCTCAAACATTAAGCGAACCAATACCATGGTCATTTTTATCAACCAATTACGTATGAAAATTGGTGTTATGTTTG
Cfau S    TGGGACTTCAAGCCCGACTTATGTCTCAAGCACTTAGAAAACTTACCTCAAACATTAAGCGAACCAATACCATGGTGATTTTTATCAACCAATTACGTATGAAAATTGGTGTTATGTTTG
Cnau S    TGGGGCTTCAAGCCCGACTTATGTCTCAAGTACCTAGAAAACTTACCTCAAATATTAAGCGAACCAATACCATGGTGATTTTTATCAACCAATTACGTATGAAAATTGGTGTTATGTTTG
Cste S    -GGGACTTCAAGTCCGACTCATGTTTCAAGCACTTAGAAAACTCACTTCAAATATTAAGCGAACCAGTACTATGATGATTTTTATCGACCAATTACGTATGAAAATTGGTGTTATGTTTG
Cpac S    TGGGACTTCAAGCCCGACTTATGTCTCAAGCACTTAGAAAACTTACCTCAAACATTAAGCGAACCAATACCATGGTGATTTTTATCAACCAATTACGTATGAAAATTGGTGTTATGTTTG
Cfos S    TGGGGCTTCAAGCTCGACTTATGTCTCAAGCGCTTAGAAAACTCACCTCAAACATTAAGCGAACTAACACTATGGTAATTTTTATCAACCAATTACGCATGAAAATTGGTGTTATGTTTG
Rma S     ------------------------------------------------------------------------------------------------------------------------
Ckaw S    ------------------------------------------------------------------------------------------------------------------------
Clau S    ------------------------------------------------------------------------------------------------------------------------
Ckil S    ------------------------------------------------------------------------------------------------------------------------
Csoy S    ------------------------------------------------------------------------------------------------------------------------
Vok  S    ------------------------------------------------------------------------------------------------------------------------

position                                                             1                                                           1
          9                                                          0                                                           0
          4     5         6          7         8         9         0         1         2         3         4         5         6
          456789012345678901234567---890123456789012345678901234567890123456789012345678901234567890123456789012345678901234567890
in recA                                635                                                                                        727
Cpha S    GCAATCCTGAAACCACAACTGGTG---GTAACGCGTTGAAATTTTACGCCTCAGTACGCTTAGATATTCGCCGCATTGGCACGATTAAAAAAGGCGATGAGATTTTAGGCAATGAAACAC
Cfau S    GCAATCCTGAAACCACAACTGGTG---GTAACGCGTTGAAATTTTACGCCTCAATACGCTTAGATATTCGCCGTATTGGTGCGATTAAAAAAGGCGATGAGATTTTAGGCAATGAAACAC
Cnau S    GCAATCCTGAAACCACAACTGGTG---GTAACGCGTTGAAATTTTACGCCTCAGTACGCTTAGATATTCGCCGTATTGGTGCGATTAAAAAAGGCGATGAGATTTTAGGCAATGAAACAC
Cste S    GCAATCCCGAAACCACAACTGTTGGGTGGTACCCGTTGAAATTTTACGCCTCAATACGCTTAGATATTCGCCGTATTGGTGCGATTAAAAAAGGTGATGAGATTTTAGGCAATGAAACAC
Cpac S    GCAATCCTGAAACCACAACTGGTG---GCAACGCGTTGAAATTTTACGCCTCAGTACGCTTAGATATTCGCCGTATTGGTGCGATTAAAAAAGGTGATGAGACTTTAGGCAATGAAACAC
Cfos S    GTAACCCTGAAACTACAACGGGTG---GTAATGCGTTAAAATTTTACGCCTCAGTACGCTTAGATATTCGCCGTATTGGCGCAATCAAAAAAGGCGATGAAATTTTAGGCAATGAAACAC
Rma S     ----------------------------------------------------------------------------------------------------------AGGCAATGAAATAC
Ckaw S    ------------------------------------------------------------------------------------------------------------------------
Clau S    ------------------------------------------------------------------------------------------------------------------------
Ckil S    ------------------------------------------------------------------------------------------------------------------------
Csoy S    ------------------------------------------------------------------------------------------------------------------------
Vok  S    ------------------------------------------------------------------------------------------------------------------------

position  1                                                                                                                      1
          0                                       1                                                                              1
          6         7         8         9         0         1         2         3         4         5         6          7       7
          123-4567890123456789012345678901234567890123456789012345678901234567890123456789012345678901234567890-123456789012345678
in recA                                                                                                                 828        839   845
Cpha S    GCG-TTAAGGTTTTAAAAAATAAAGTTGCACCGCCCTTCAAACAAGCAGAATTTCAAATCCTTTACAATGAGGGTATTTCACTTGAAAGTGAAATAATTGA-TTTAGGCGTGAAGCACGG
Cfau S    GCG-TTAAAGTATTAAAAAATAAAGTTGCATCACCCTTCAAACAAGCAGAATTTCAAATCCTTTACAACGAGGGTATTTCACTTGAAAGTGAAATAATTGA-TTTAGGCGTGAAGCACGG
Cnau S    GCG-TTAAAGTATTAAAAAATAAAGTTGCACCACCCTTCAAACAAGCAGAATTTCAAATCCTTTACAACGAGGGTATTTCACTTGAAAGTGAAATAATTGATTTTAGGCGTGAAGCATGG
Cste S    GCG-TTAAAATATTAAAAAATAAAGTTGCACCACCTTTCAAACAAGCAGAATTTCAAATCCTTTACAATTAGGATATTTTACTTGAAAGTGAAATAATTGA-TTTAG------AGCACGG
Cpac S    GCG-TTAAAGTATTAAAAAATAAAGTTGCACCACCCTTCAAACAAGCAGAATTTCAAATCCTTTACAACGAGGGCATTTCACTTGAAAGTGAAATAATTGA-TTTAGGTGTGAAACACGG
Cfos S    GCG-TTAAAGTATTAAAAAACAAAGTCGCACCACCTTTCAAACAAGCAGAATTTCAAATTCTTTACAACGAAGGCATTTCACTTGAAAGTGAAATAATTGA-TTTAGGCGTGAAGCATGG
Rma S     GTGTTTTAAGTATTAAAAAATAAAGTCACATTACTTTTCAAACAAGCAGGATTTTAAATCCTTTATAAATAGGAGATTTCACTTGAAAGTGAAATAATTGA-TTTGAGTATGAAGCTTT- 695
Ckaw S    ------------------------------------------------------------------------------------------------------------------------
Clau S    ------------------------------------------------------------------------------------------------------------------------
Ckil S    ------------------------------------------------------------------------------------------------------------------------
Csoy S    ------------------------------------------------------------------------------------------------------------------------
Vok  S    ------------------------------------------------------------------------------------------------------------------------

position  1                                                                                                                      1
          1                    2                                                                                                 2
          78         9         0         1         2         3         4         5         6          7         8         9       9
          9012345678901234567890123456789012345678901234567890123456789012345678901234567890-1234567890123456789012345678901234567
in recA                                                                                                                           964
Cpha S    GTTTGTTGAAAAAGCAGGTGCTTGGTATAGTGTTGAAGGGGAACGAATTGGCCAAGGTAAAGACAACGCTCG-TGAATATTTAAAATTAAACCCTAAGCTCAGTCAAAAAATAGAGACAC
Cfau S    GTTTGTTGAAAAAGCAGGCGCTTGGTATAGTGTTGAAGGAGAGCGAATTGGCCAAGGTAAAGATAACGCTCG-TGAATATTTAAAATCAAACCCTAAGCTCAGTCAAAAAATCGAGATAC
Cnau S    GTTTGTTGAAAAGCAGGTGCTTGGTATAGTGTTGAAGGGGAGCGAATTGGCCAAGGTAAAGACAACGCCCG-TGAATATTTAAAATCAAACCCTAAGCTCAGTCAAAGAATAGAGACAC
Cste S    GTTTGTTGAAAAAGCAGGTGCTTGGTATAGTATTGAAGGGGAGTGAATTTGCCAAGGTAAAGACAACGCCCG-TGAATATTTAAAATCAAACCCTAAGCTCAGTCAAAAAATAGAGACAC
Cpac S    GTTTGTTGAAAAAGCAGGTGCTTGGTATAGTGTTGAAGGAGAGCGAATTGGCCAAGGTAAAGACAACGCCCG-TGAATATTTAAAATCAAACCCTAAGCTCAGTCAAAAAATCGAGATAC
Cfos S    CTTTGTCGAGAAAGCAGGTGCTTGGTATAGCGTTGAAGGGGAGAGAATTGGTCAAGGTAAAGACAACGCTCG-TGAATATTTAAAAGCTAACCCTGAGCTTAGTCAAAAAATTGAGACAC
Rma S     -----------------------------------------------------------------ATGCTCATTAAATATTC---------TTTTTAGATTGG-----------------
Ckaw S    ------------------------------------------------------------------------------------------------------------------------
Clau S    ------------------------------------------------------------------------------------------------------------------------
Ckil S    ------------------------------------------------------------------------------------------------------------------------
Csoy S    ------------------------------------------------------------------------------------------------------------------------
Vok  S    ------------------------------------------------------------------------------------------------------------------------
```

```
position  1                                                                                                       1              1
          2 3                                                                                                     4              4
          9 0        1         2         3          4         5          6         7         8         9         0         1    1
          890123456789012345678901234567890123456-78901234567890-1234567890123456789012345678901234567890123456789012345678901234567890123456789012345
in recA                                         1004           1018                                                                  1082
Cpha S    AAATCCGTGAAAAAATTACTAACTAAAGAAGCAGATATTG-AAAAGGACGTTAGC-AATCAAGACGTCGATTAACCAAGCTTACCACCAGGTGTTAAGCTTACTGGTTCAGCGTGAGCATT
Cfau S    AAATCCGTGAAAAAATTACTAACTAAAGAAGCAGATATTGAAAAAGGACGTTAGC-AATCAAGACGTCGATTAATCAAGCTTATCACCAGATTTTAAGTTTATTGGCCCAGCGTGAGTATT
Cnau S    AAATCCGTGAAAAAATTACTAACTAAAGAAGCAGATATTG-AAAAGGACGTTAGC-AATCAAGACGTCGATTAATCAGGCTTATCACCAGGTCTTAAGTTTATTGGCTCAACGTGAGCATT
Cste S    AAATCCGTGAAAAAATTACTAACTAAAAAAGCAGGTATTG-AAAAGGACGTTAGCAAATCAAGACGTTGATTAATCAGGCTTATCACCAGGTCTTAAGTTTATTGGCTCAGCGTGAGCATT
Cpac S    AAATTCGTGAAAAAATTACTAACTAAAGAAGCAGATATTG-AAAAGGACGTTAGC-AATCAAGACGTCAATTAATCAGGCTTATCACCAGGTCTTAAGTTTATTGGTCCAGCGTGAGCATT
Cfos S    AAATTCGTGAAAAAATTACTAGCTAAAGAGTCAGGTATTG-AAAAGGACGTTAGC-AATCAAGACACCAACTAATCAAGTTTACCAGCAGGCATTAAGTTTACTGGCTCAGCGTGAGCATT
Rma  S    ---TGTTTGGAAAATCATTGTTTATATAG-----------------------------------------------GTTT----------ATTGATTTTTGTGGTTAAGCATGTCCAAA 783
Ckaw S    ----------------------------------------CATGGAATATTGTG------------------------------------------------------------------------
Clau S    ----------------------------------------AATAGAGTATTGGG------------------------------------------------------------------------
Ckil S    ----------------------------------------AATAGAGTATTGGG------------------------------------------------------------------------
Csoy S    ----------------------------------------AATAGAGTATTGGG------------------------------------------------------------------------
Vok  S    ----------------------------------------AATAGAGTATTGGG------------------------------------------------------------------------

position  1                                                                                                                       1
          4                                                                                         5                             5
          1   2         3         4         5         6         7         8         9         0         1         2         3    3
          678901234567890123456789012345678901234567890123456789012345678901234567890123456789012345678901234567890123456789012345

Cpha S    CTGCTTTAGAGTTGACACGAAAACTAAGCGCCAAAGGTTATGTCGTCGAAGAAATAGAGCCAGTCTTAGCGCAACTAGCACAAAATAATTATCAAAGCGATGAGCGCTTTGCTGAGGCAT
Cfau S    CTGCTTTAGAGTTGACGCAAAAATTAAGTACTAAAGGTTATGCTATTGAAGAAATAGAGTCAGTATTAGTGCAACTAGCACAAAATAATTATCAAAGCGATGAACGCTTTTCTAAGGCGT
Cnau S    CTGTTTTAGAGTTGACGCAAAAACTAAGTGCTAAAGGTTATGCTATTGAAGAAATAGAGCCAGCATTAGTGCAACTAGCACAAAATAATTATCAAAGCGATGGACGCTTTGTTGAGGCGT
Cste S    TTGCTTTAGAGTTGACGCAAAAACTAAGTGCTAAAGGTTATGCTATTGAAGAAATAGAGCTAGCATTAGTGCAACTAGCACAAAATAATTATCAAAGCGATGAACGCTTTGCTGAGGCGT
Cpac S    CTGCTTTAGAGTTGACGCAAAAACTAAGTGCTAAAGGCTATGCTATTGAAGAAATAGAGCCAGCATTAGTGCAACTAGCACAAAATAATTATCAAAGCGATGAACGTTTTGCTGAAGCGT
Cfos S    CTGCTTTAGAACTGACACGAAAACTAAGCGCCAAAAGTTATGCTGTTGAAGAAATAGAGCCAGCTTTAGTGCGACTAGCACAAAATAATTATCAAAGCGATGAGCGCTTTGCTGAGGCAT
Rma  S    ATATTTAAAAAATATACAC------------------------TTATTAAGAAATTGTATTCATTATT---------------------TTATTTATACACTGAGC----------GGCAT
Ckaw S    ------------------------------------------------------------------------------------------------------------------------
Clau S    ------------------------------------------------------------------------------------------------------------------------
Ckil S    ------------------------------------------------------------------------------------------------------------------------
Csoy S    ------------------------------------------------------------------------------------------------------------------------
Vok  S    ------------------------------------------------------------------------------------------------------------------------

position  1                                                                                                                       1
          5                                                                                 6                                     6
          3   4         5         6         7         8         9         0         1         2         3         4         5    5
          678901234567890123456789012345678901234567890123456789012345678901234567890123456789012345678901234567890123456789012345

Cpha S    TTGTACTTATGCGCACTAATCAAGGCAAGGGCGGTGTGCTAATCAGCCAACAACTCAAACAAAAAGGCATTGAAGATTTTGATTTATCAACCATTGATTTTTTTGAACTTGCTAAAAGCG
Cfau S    TTGTACTTATGCGCACTAATCAAGGTAAGGGTGATGTATTAATCAGTCAACAATTGAAACAAAAAGGTATTGAAGATTTTGATTTATCAACCATTGATTTTTTTGAACTTGCTAAAAGGG
Cnau S    TTGTACTTATGCGCATTAATCAAGGTAAGGGTGGTGTATTAATCAGTCAACAATTGAAACAAAAAGGTATTGAAGATTTTGATTTATCAACCATTGATTCTTTTGAACTTGCTAAAAGCG
Cste S    TTGTACTTATGCGCACTAATCAAGGCAAGGGTGGTGTATTAATCAGTCAACAATTGAAACAAAAAGGTATTAAAGATTTTGATTTATCAACCATTGATTTTTTTGAACTTTCTAAAAGCG
Cpac S    TTGTACTTATGCGTACTAATCAAGGTAAGGGTAGTGTATTAATCAGCCAACAATTGAAACAAAGAGGTATTGAAGATTTTGATTTATCAACCATTGATTTTTTTGAACTTGCTAAAAGCG
Cfos S    TTGTACTTATGCGTACTAATCAAGGCAAGGGTAGTGTGCTAATCAGCCAACAACTCAAACAAAAAGGCATTGAAGATTTTGATTTATCAACCATTGATTTTTTTGAACTTGCTAGTAATG
Rma  S    ------------GTAATAATCAA------------------------------------------------------------------------------------------------
Ckaw S    TTGTAATTATATCTATCTAGTTAGATA----------------------------------------------------------------------------------------------
Clau S    TTGTAATTATACCTATCTAGGTAGTTA----------------------------------------------------------------------------------------------
Ckil S    TTGTAATTATACGTACCTAGTTAGTTA----------------------------------------------------------------------------------------------
Csoy S    TTGTAATTATACGTACCTAGTTAGTTA----------------------------------------------------------------------------------------------
Vok  S    TTGTAATTATACGTACCTAGTTAGTTA----------------------------------------------------------------------------------------------
                         *   *   *

position  1                                                                                                                       1
          6                                                    7                                                                  7
          5   6          7         8         9         0         1         2         3         4         5         6         7    7
          6789012-345678901234567890123456789012345678901234567890123456789012345678901234567890123456789012345678901234567890123456789012345678901234

Cpha S    TTAGACT-AAAAAAATATGGCGAGCAATTGCCTAAAAATTATAAAGAAAAAGCCAAACAACAGCGTTTTTTACAATCACGTGGGTTTGGTTTTGAACAAATTAACCAAGTATTAGTTGT
Cfau S    TTAGATT-AAAAAAATATGGCGAGCAATTACCTAAAGATTATAAAGAAAAAGCTAAACAACAGCGTTTTTTACAATCACGTGGGTTTGATCTTGAGCAAATTAACCAAGTATTAGCTGT
Cnau S    TTAGACT-AAAAAAATATGGCAAGCAATTGCCTAAAGATTATAAAGAAAAAGCCAAACAACAGCGTTTTTTACAATCACGTGGGTTTGATTTTGAACAAATTAACCAAGTATTAGTTGT
Cste S    TTAGATTAAAAAAAATATGGCGAGCAATTTCCTAAAAATTATAAAGAAAAAGCCA-------------------------------------------------------------
Cpac S    TTAGACT-AAAAAAATATGGCGAGCAATTGCCTAAAAATTATAAAGAAAAAATCAAACAACAGCGTTTTTTACAATCACGTGGGTTTGATTTTGAGCAAATTAACCAAGTATTAGTTGT
Cfos S    TACGAGT-AAAAAAATATGGTGAACAATTGCCTAAAAATTATAAAGAAAAAGCCAAACAGCAACGTTTTTTGCAATCTAGAGGCTTTGGATTTGATGAAATTAATCAAGCGTTCAATTGT
Rma  S    -----------------------------------------------------------------TCTTTTACAAACTAGGAATTTTTAGATTTTTGCTATTAATTG-------------
Ckaw S    ------------------------------------------------------------------------------------------------------------------------
Clau S    ------------------------------------------------------------------------------------------------------------------------
Ckil S    ------------------------------------------------------------------------------------------------------------------------
Csoy S    ------------------------------------------------------------------------------------------------------------------------
Vok  S    ------------------------------------------------------------------------------------------------------------------------

position  1                                                                                                                       1
          7                        8                                                                                              8
          7    8         9         0         1         2         3         4         5         6         7         8         9    9
          567890123456789012345678901234567890123456789012345678901234567890123456789012345678901234567890123456789012345678901234

Cpha S    GAATAAACTATTTACTATGCGCTCACCTTTGTATATTAAATACCCTAATGGCGAAACAAGAGTAATAGAGGCTTTGTTTGAATATCTAAAGGGTGTTTTATATTTGAATTTTTTTTAGA
Cfau S    GAATAGACTATTTACCATGCACTCACCTTTGTATATTAAATATCCTTATAGTGAAATAAGAGTAATAGAGGCTTTATTTGAA---------------------------------------
Cnau S    GAATGAACTATTTACTATGCGCTCACCTTTGTATATTAAATTTCCTAATGCTGAAACCAGAGTAGTAGAGGCTTCATTTTAACATCTAAAGGGTGTTTTATATTTGAACTTTTTTGGGA
Cste S    -------------------------------------------------AATGGTGAAACAGAGTAATAGAGGCTTTATTTGAACATCCAAAGGGTGTTTTATATTTGAACTTTTTTGGGA
Cpac S    GAATAAACTATTCACCATGCGTCCACCTTTGTATATTAAATATCCTAATAGTGAAATAAGAATAATAGAGGCTTTATTTGAACA--------------------------------------
Cfos S    GAATAAACTATTCACCATGCAATCTCCTTTGTATATCAAATACCCTAATGGTGAAACAAGAGTTATAGAAGCCTTATTTGAACATCCAGAGGGTATTTTGTATTTTGAACTTTTTTTGGA
Rma  S    --------CATTTATTATCATATTTAGTGTATTTTTCATTAACGCCAAT---------AAAATTACAAA----TTATGTAA------------TATACTGAATATTAA--TTTTATTAGA 986
Ckaw S    ------------------------------------------------------------------------------------------------------------------------
Clau S    ------------------------------------------------------------------------------------------------------------------------
Ckil S    ------------------------------------------------------------------------------------------------------------------------
Csoy S    ------------------------------------------------------------------------------------------------------------------------
Vok  S    ------------------------------------------------------------------------------------------------------------------------
```

```
position  1                                                                                                         2            2
          8    9                                                                                                    0            0
          9    0         1         2         3         4          5         6         7         8         9         0         1  1
          5678901234567890123456789012345678901234567890123-4567890123456789012345678901234567890123456789012345678901234567890123

Cpha S    AAAAGACCTAACTTATAGTATTAATATTATTAAAGGCGAGGTAATGGGC-GAAGGGCCTTGGAAAGTTGGCCAGTGCTCGTTACACGTATTGGGTTGTAATCATACGCATCCACAGTTGT
Cfau S    ------------------------------------------------------------------------------------------------------------------------
Cnau S    AAAAGACCCAACTTATAGTATTCATATTATCAAAGACGAGGTAATGGGT-GAAGGGCCTTGGAGAGTTGGCTAGTGCTCGTTACACGTATTAGGTTGTAATCATACACATCCACAGTTGT
Cste S    AAAAGACCCAACTTATAGTATTTATATTATCAAAGGGGGGGTAATGGGCGGGAGGGCCTTGGAGAGTTGGCCAGTGCTCGTTACACGTATTAGGTTGTAATCATACACATCCACAGTTGT
Cpac S    ------------------------------------------------------------------------------------------------------------------------
Cfos S    AAAAGACCCAACTTATAGTATTCATATTATTAAAGACGAGGTGATGGGCG-ATGGTCCTTGGAAAGTTGGGCAGTGTTCGTTACACGTATTGGGGTGTAATCATACACACCCACAGTTGT
Rma S     --------------------ATTCTTATTTTTA--------------------------------------TAGCGTTC-----------------ATAATGACGCATACTCA-------
Ckaw S    ------------------------------------------------------------------------------------------------------------------------
Clau S    ------------------------------------------------------------------------------------------------------------------------
Ckil S    ------------------------------------------------------------------------------------------------------------------------
Csoy S    ------------------------------------------------------------------------------------------------------------------------
Vok  S    ------------------------------------------------------------------------------------------------------------------------

position  2                                                                                                                     2
          0                                                                                     1                               1
          1     2         3         4         5         6         7         8         9         0         1         2         3 3
          45678901234567890123456789012345678901234567890123456789012345678901234567890123456789012345678901234567890123456789012

Cpha S    GTGAGATGCATGCGTTCTGGCAACAAGAATTATTGCAAAATCTAGCTCAGTTTAGAGCTAGTGATGTGGTTAAAATTGCTTGTGAAAAAGGTGCCTTTTTACCTGATAACCGCCCAATT-
Cfau S    --------------------------------------------------------------------------------------------------------------CACCCAATTA
Cnau S    GTGAGATACATGCGTTTTGGCAACAAGAATTATTGTAAAATATAGCTCAATTTAGAGCTAATGATATGGTGAAAATTGCTTGTGAAAAAAGCACCTTTTTACCTGATAAACACCCAATT-
Cste S    GTGAGATGTGTACGTTTTGGTAACAAGAATTATTGCAAAATCCAGCTCAATTTAGAGCCAGTGATATGGTGAAAATTGCTTGTGAAAAAAGTGCCTTTTTACCTGATAAACACCCAATT-
Cpac S    ----------------------------------------------------------------------------------------------------------------CCCAATTA
Cfos S    GTGAAATGCGTGCATTTTGGCAACAAGAATTATTGCAAAATCCAGACCAGTTTAGAGCCAATGACATGGTGAAAATTGCTTGTAAAAAAAGTGCGTTTTTACCCGGTGATTACCCGATA-
Rma S     ---AGATGTAAAAATTATGA----AGAAAATATTGTGCAA-----------AGCGTTTATG--ATTACGAGATGTATGAGCAAGATAAAGGTGTATTTTTATCCGATA------------
Ckaw S    ----------TGTATATTAGTAACAAGAATTATTAGAGAATTAAGATTAGTTTAGAATCAATGAGATGATAAAAATTACTTGTAAAA---GTGTACCTTTGCTTAATAATAATTTTATA-
Clau S    ----------TGTATCTTGGCAACAATAATTATTACAGAATCCAGATTAGTTTAGAACCAATGAGATGATAAAAATTACTTGTAAAAAAGGCTTACTTTTGCCTAATAATAATCTTATA-
Ckil S    ----------TGTATCTTGGTAATAAGAATTATTACAGAATCCAGATTAGTTTAGAACCAATGAGATGATAAAAATTACTTGTAAAAAAGGCGTACCTTTACCCAATAATAATCTTATA-
Csoy S    ----------TGTATCTTGGTAATAAGAATTATTACAGAATCCAGATTAGTTTAGAACCAATGAGATGATAAAAATTACTTGTAAAAAAGGCGTACCTTTACCCAATAATAATCTTATA-
Vok  S    ----------TGTATCTTAGTAATAAGAATTATTACAGAATCCAGATTAGTTTAGAACCAATGAGATGATAAAAATTACTTGTAAAAAAGGCGTACCTTTACCCAATAATAATCTTATA-
                                                                                                                              **

position  2                                                                                                                      2
          1                                                                   2                                                  2
          3      4         5         6         7         8         9          0         1         2         3         4          5
          34567890123456789012345678901234567890123456789012345678901234567-890123456789012345678901234567890123456789012345678901

Cpha S    AATGATGCGCGGTAATGATAAACAAACCCAAGCCTATGATTTTAAAGCCAGAGATGTTAAGGTTTT-AAAAAAGCTTTTGCCTTATTTATTAAAAATGCGTGTGCGCGTTGTTTTGGCAA
Cfau S    AATGATGCGTGG-AC----------------------------------AGAGATCTTAAGGTTTTAAAAAAAGCTTTTGCCTTATTTATTAAAAATGCGTGTGCGCGTTCTTTTGGCAA
Cnau S    AATGATGTTCGGTACTGATAAACAAACCCAAACCTATGA---TAAAACCAGAGATGTTAAGGTTTT-AAGAAATATTTTGCCTTATTTGTTAAAAATGCGTGTGCGTGTTGTTTTGGCAA
Cste S    AATGATTCGCGGTACTGATAAACAAACCCAAGCCTATGGTTTTAAAACTAGAGATGTTAAGGTTTT-AAAAAAGCTTTTGCCTTATTTATTAATAATACGTGTGTTCGTTGTTTTGGCAA
Cpac S    AATGATGCGTGGTACTGATAAACAAACCTAAACCTATGATTTTAAAACTAGAAGTTTTAAGGTTTT-AAAGAAACTTTTGCCTTATTTATTAAGAATGCATATGTGCGTTGTTTTGGCAA
Cfos S    AACGATGCGCAGTACTGATAAACAAACCAAGGCTTATGATTTTAAAACCAGAGATATTAAGGTTTT-AAAAAAGCTCCTACCTTATTTACTAAAAATGCGTGTGCGTGTTATTTTGGCAA
Rma S     AATGATGCGTAGTACTGATAAACAAACCAAGGCTTATAATTTTAAAACTAGAGATATTAAAGTTTT-AAAAAAGCTCCTACCTTATTTACTAAAAATGCGTGTGCGTGTTATTTTGGCAA
Ckaw S    CACTATGTGTAGCACTGATAAGCAAACTAAGGCTTATGATTTTGAAACCAGAGACATTAAGGTTTT-AAAAAAGTTATTCCCTTATTTGTTAAAAATGCGTATACGTGTTATTTTGGCAA
Clau S    CACTATGTGTAGTACTGATAAGAAAACTAAGGCTTATGATTTTAAAACCAGAGACATTAAGGTTTT-AAAAAAGTTATTTCCTTATTTGTTAAAAATGCGTATACGTGTTATTTTGGCAA
Ckil S    CACTGTGTGTAGTACTGATAATCAAACTAAGGCTTATGATTTTAAAACCAGAGACATTAAGGTTTT-AAAAAAGTTATTTCCTTATTTGTTAAAAATGCGTATACGTGTTATTTTAGCAA
Csoy S    CACTGTGTGTAGTACTGATAATCAAACTAAGGCTTATGATTTTAAAACCAGAGACATTAAGGTTTT-AAAAAAGTTATTTCCTTATTTGTTAAAAATGCGTATACGTGTTATTTTAGCAA
Vok  S    CACTGTGTGTAGTACTGATAAGCAAACTAAGGCTTATGATTTTAAAACCAGAGATATTAAGGTTTT-AAAAAAGTTATTTCCTTATTTGTTAAAAATGCGTATACGTGTTATTTTGGCAA
           *   *                                           ***    **** ***** **  **  *  * ********  *** *** * * *    *** **** ****

position  2                                                                                                                      2
          2                                                3                                                                     3
          5       6         7          8         9         0         1         2         3         4         5         6         6
          234567890123456789012345-67890123456789012345678901234567890123456789012345678901234567890123456789012345678901234567-89

Cpha S    CAATGTTTTTAATTTTCGCAAAGC-TTGCCAATGTTACAGTACCCGTTGTTTTAAAAGAAATTGTTGATTCTTTAGATCAAACTAACCTTTTACTAATTTTGCCATTAGGGCTACTT-TT
Cfau S    TAATGTTTTTAATTTTCGAAAAAC-TTGCTAATGTTGCAGTGCCTGTTGTTTTAAAAGAAATTGTTGATTCTTTAGAGCAAACTAACTTATTGCTGATTTTGTCATTAGGGTTGCTTCTT
Cnau S    CAATGTTTTTAATTTTCGAAAAAC-TTGTCAATGTTACAGTACCCGTTGTTTTAAAAAAAATTGTTGATTCTTTAGACCAAACTAACCTATTACTGATTTTTTCATTAGGATTACTC-TT
Cste S    CAATGTTTTTAATTTTCGCAAAAC-TTGCCAATGTTGCAGTGCCCGTTGTTTTAGAAGAAATTGTTGATTATTTAGACCAAACTAACCTATTACTGATTTTGCCATTAGGATTGCTC-TT
Cpac S    CAATATTTTTAATTTTCGCAAAACATTGCCAATGTTGCAGTGCCTGTTGTTTTAAAAGAAATTGTTGATGCTTTAGAGCAAACTAACCTATTACTGATTTTGCCATTAGAACTGCTC-TT
Cfos S    CGTTATTTTTAATTGCTGCAAAGT-TTGCTAATGTTGCAGTGCCTGTTGTTTTAAAAGAAATTGTTGATTCTTTAGATCAAACTAACCTTTTACTGATTTTGCCATTAGGGTTGCTT-TT
Rma S     CGTTATTTTTAATTGCTGCAAAGC-TTGCTAATGTTGCAGTACCTGTTGTTTTAAAAGAAATTGTTGACTCTTTAGATCAAACTAATCTTTTACTGATTTTACCATTAGGGTTGCTT-TT
Ckaw S    TGTTATTTTTGATTTTTGCAAAAT-TTGCTAATGTTGCAGTGCCAGTTGTTTTAAAAGAAATCGTTGATGTCTTAAATCATACTAATATCTTGCTAATTTTACCGTTAGGACTACTT-TT
Clau S    TGTTATTTTTGATTGTTGCAAAAT-TTGCTAATGTTGCAGTGCCAGTTGTTTTAAAAGAAATCGTTGACGTCTTAAATCAGACTAACATCTTGTTAATTTTACCGTTAGGACTACTT-TT
Ckil S    TGTTATTTTTGATTGTTGCAAAAT-TTGCTAATGTTGCAGTACCAGTTGTTTTAAAAGAAATCGTTGACGTCTTAAACCAGACTAATATCTTACTAATTTTACCGTTAGGACTACTT-TT
Csoy S    TGTTATTTTTGATTGTTGCAAAAT-TTGCTAATGTTGCAGTACCAGTTGTTTTAAAAGAAATCGTTGACGTCTTAAACCAGACTAATATCTTACTAATTTTACCGTTAGGACTACTT-TT
Vok  S    TGTTATTTTTGATTGTTGCAAAAT-TTGCTAATGTTGCAGTGCCAGTTGTTTTAAAAGAAATCGTTGACGTCTTAAACCAGACTAATGTCTTATTAATTTTACCGTTAGGACTACTT-TT
             * ***** ***   * ***   ***  ****** **** ** ********* ** **** *****    *** * ** *****  * **  * *****  * ****   * **  **

position  2                                                                                                                      2
          3                               4                                                                                      4
          7         8           9         0         1         2         3            4         5          6         7          8 8
          01234567890123--456789012345678901234567890123456789012345678901234567---89012345678901234-567890123456789012345-6789012

Cpha S    TGCTTATGGCGTAC--TTAGGCTTGCTAGTTCGTTGTTTAACGAACTCCGTGATGCGATTTTTGCACGCG---TTCGTTATCATGCGATG-CATTTGATTGCACTTGGTGTA-TTTAAGC
Cfau S    TGCCTATGGTGCACCATAAGGCTTGCTAGTTTGTTATTTAATGAGCTTCGTGATACGATTTTTGCGTGCG---TCCGTTATCACGCTATG-TATTTGATTGCACTTGCTGTG-TTTAAGC
Cnau S    TGCCTATGGTCC----TTAGGCTTGCTAGTTCGTTATTTAATGAGCCTCGTGATACGATTTTTGTGTACG---TCCGTTATCAGGCTATA-CATTTGATTGCACTTGGTGTGTTTTAAGT
Cste S    TGCCTATGGTGCAT--TTAGGCTTGCTAGTTCGTTATTTAATGAGCTTTGTTATACGATTTTTGCGCGCG---TTCG-TATCACGCTATG-CATTTGATTGCACTTGGTGTG-TTTAAGC
Cpac S    TGCCTATGGTGCAC--TTAGACTTGCTAGTTTGTTATTTAATGAGCTTCGTGATGCGATTTTTGCGCGCGGGTTCCGTTATCATGTTATATTATGCGATTGCACTTGGTGTG-TTTAAGC
Cfos S    TGCTTATGGTGCGC--TTAGGCTTGCTAGTTCGTTGTTTAACGAGCTTCGTGATGCGATTTTTGCGCGCG---TTCGTTATCATGCGATG-CATTTAATTGCACTTGGTGTA-TTTAAGC
Rma S     TGCTTATGGTACGC--TTAGGCTTGCTAGTTCGTTATTTAATGAGTTTCGTGATGCGATTTTTGCGCGCG---TTCGTTATCATGCGATG-CATTTGATTGCACTTGGTGTA-TTTAAGC
Ckaw S    TGCTTACGGCGCAC--TTAGACTTGCTAGTTCGTTATTTAACGAGCTTCGTGATGCGATTTTTGTACGTG---TTCGTTATCATACTATG-CGTTTGATTGCAATTAGTGTA-TTTAAGC
Clau S    TGCTTACGGTGCAC--TTAGACTTGCTAGTTCGTTATTTAACGAGCTTCGTGATGCGATTTTTGTACGTG---TTCGTTATCATACTATG-CGTTTGATCGCAATTAGTGTA-TTTAAGC
Ckil S    TGCTTACGGTGCAC--TTAGACTTGCTAGTTCGTTATTTAACGAGCTTCGTGATGCGATTTTTGTACGTG---TTCGTTATCATATTATG-CGCTTGATTGCAATTAGTGTA-TTTAAGC
Csoy S    TGCTTACGGTGCAC--TTAGACTTGCTAGTTCGTTATTTAACGAGCTTCGTGATGCGATTTTTGTACGTG---TTCGTTATCATATTATG-CGCTTGATTGCAATTAGTGTA-TTTAAGC
Vok  S    TGCTTACGGTGCGC--TTAGACTTACTAGTTCGTTATTTAACGAGCTTCGTGATGCGATTTTTGTACGTG---TTCGTTATCATATTATG-CGTTTGATTGCAGTTAGTGTA-TTTAAGC
          *** ** **       * ** *** ****** *** ***** **     ** ** *********     *   * ** *****    **        ** *** **  ***  ******
```

```
position  2                                                                                                                      2
          4               5                                                                                                      5
          8    9          0         1         2         3         4          5         6         7             8         9    9
          3456789012-345678901234567890123456789012345678901234567890123-45678901234567890123456789--012345--67890123456789012-345

Cpha S    ATTTACACAC-ATTGGACTTGTCTTTTCATTTGGATCGCCGTATTGGCGGTATTACTCGTGA-TATTGACCGTGGCACTCAAAGTGTAT--CCACCT--TATTATCGATTTTTGTG-TTT
Cfau S    ACTTGTATAC-ACTGGGTTTATTTTTTCATTTAGATTGCCGCAATGGTGGAATTACTTGTGATTATTAGTCGTTGCACTCAAAGTATGT--CTACCCTGTGTTGTCGATTTT-----TTT
Cnau S    ACTTGCATAT-ACTGGATTTATCTTTTTATTTAGATCACCGTATTGGTGAGATTACTCGTGA-TATTGATCGTGGCATTCAAAGTG-----CTACCC--TGTTGTCTATTTTTTTG-TTT
Cste S    ACTTGCATAC-ACTGGATTTATTTTTTCATTTAGATTGCCGCATTGGTGGGGTTACTCGTGA-TATTGATCGTGGCACTCAAAGTGTGTC-CTACCC--TGTTGACGGTTTTTTTTGTTT
Cpac S    ACTTGCATACAACTGGATTTATCTTTTCATTTAGATCGCCGCATTGGTGGAATTACTCGTGA-TATTGATCGTGGCACTCAAAGTGTCTCTCTACCC--TGTTGTCAATTTT-----TTT
Cfos S    ATTTACACAC-ACTGGACTTGTCTTTTCATTTGGATCGCCGTATTGGTGGTATTACTCGTGA-TATTGATCGCGGCACTCAAAGTGTGT--CCACCT--TATTATCGATTTTTGTG-TTT
Rma S     ATTTACACAC-ACTGGATTTGTCTTTCCATTTGGATCGTCGTATTGGTGGTATTACTCGTGA-TATTGACCGCGGTACTCAAAGTGTAT--CGACCT--TGTTGTCGATTTTTGTG-TTT
Ckaw S    ATTTACATAC-ATTGGATTTGTCTTTCCATCTGGATCGTCGTATTGGGGGTATTACCCGTGA-TGTTGACCGTGGTACTCAAAGTGTAT--CTACCT--TGTTATCGATTTTTGTA-TTT
Clau S    ATTTACATAC-ATTGGATTTGTCTTTCCATCTGGATCGTCGTATTGGGGGTATTACCCGTGA-TGTTGACCGTGGTACTCAAAGTGTAT--CTACCT--TGTTATCGATTTTTGTG-TTT
Ckil S    ATTTACATAC-ATTGGATTTGTCTTTTCATCTGGATCGTCGTATTGGTGGTATTACCCGTGA-TGTTGACCGTGGTACTCAAAGTGTAT--CTACCT--TGTTATCGATTTTTGTG-TTT
Csoy S    ATTTACATAC-ATTGGATTTGTCTTTTCATCTGGATCGTCGTATTGGTGGTATTACCCGTGA-TGTTGACCGTGGTACTCAAAGTGTAT--CTACCT--TGTTATCGATTTTTGTG-TTT
Vok  S    ATTTACATAC-ATTGGATTTGTCTTTTCATCTAGATCGTCGTATTGGTGGTATTACCCGTGA-TGTTGACCGTGGTACTCAAAGTGTAT--CTACCT--TGTTATCGATCTTTGTG-TTT
          * **  * *  * ***  ** * ***  ** * ***   ** * *** *   ****  **** * **   **  * * *******      * ***   * **  *  * **     ***

position  2                                                                     2
          5   6                                                                 6
          9   0         1         2         3         4         5         6    6
          67890123456789012345678901234567890123456789012345678901234567890123456

Cpha S    AACATTATTCCTTCATTTTTTGAGATATGTTTAGTGATTGGTATTTTGTGGCTGAATTATGATATTTTCTT
Cfau S    AACATTATTCCTTCGTTTTTTGAGATATGTTTAGTGATTGGTATTTTGTGGCTGAATTATGATATTTTCTT
Cnau S    AACATTATTCCTTCGTTTTTTGAGATATGTTTAGTGGTTGGTGTTTTGTGACTGAATTATGATATTTTCTT
Cste S    AACATTATTCCTTCGTTTTTTGAGATATGTTTAGTGATTGGTATTTTGTGGCTGAATTATGATATTTTCTT
Cpac S    AACATTATTCCTTCG-TTTTTGAGATATGTTTAGTGATTAGTATTTTGTGTCTGAATTATGATATTTTCTT
Cfos S    AACATTATTCCTTCGTTTTTTGAGATATGTTTAGTGATTGGTATTTTGTGGCTAAATTATGATATTTTCTT
Rma S     AATATTATTCCTTCATTTTTTGAGATATGCTTAGTAATTGGTATTTTGTGGCTGAATTATGATTTTTTTTT
Ckaw S    AACATCATTCCTGCATTTTTTGAGATATGTTTAGTGATTGGTGTTTTGTGGTTTAATTATGATATTTTCTT
Clau S    AACATAATTCCTGCATTTTTTGAGATATGTTTAGTGATTGGTGTTTTGTGGTTTAATTATGATATTTTCTT
Ckil S    AACATCATTCCTGCATTTTTTGAGATATGTTTAGTAATTGGTGTTTTGTGGTTTAATTATGATATTTTTTT
Csoy S    AACATCATTCCTGCATTTTTTGAGATATGTTTAGTAATTGGTGTTTTGTGGTTTAATTATGATATTTTTTT
Vok  S    AACATCATTCCTGCATTTTTTGAGATATGTTTAGTGATTGGTGTTTTGTGGTTTAATTATGATATTTTCTT
          ** ** ****** *  ************* *****  ** ** *******  * ********* **** **
```
