# Supplementary figures and images for: Loss of genes for DNA recombination and repair in the reductive genome evolution of thioautotrophic symbionts of Calyptogena clams
Source: BMC Evol Biol. 2011 Oct 3;11:285. doi: 10.1186/1471-2148-11-285 (PMC3202245; doi:10.1186/1471-2148-11-285)

A

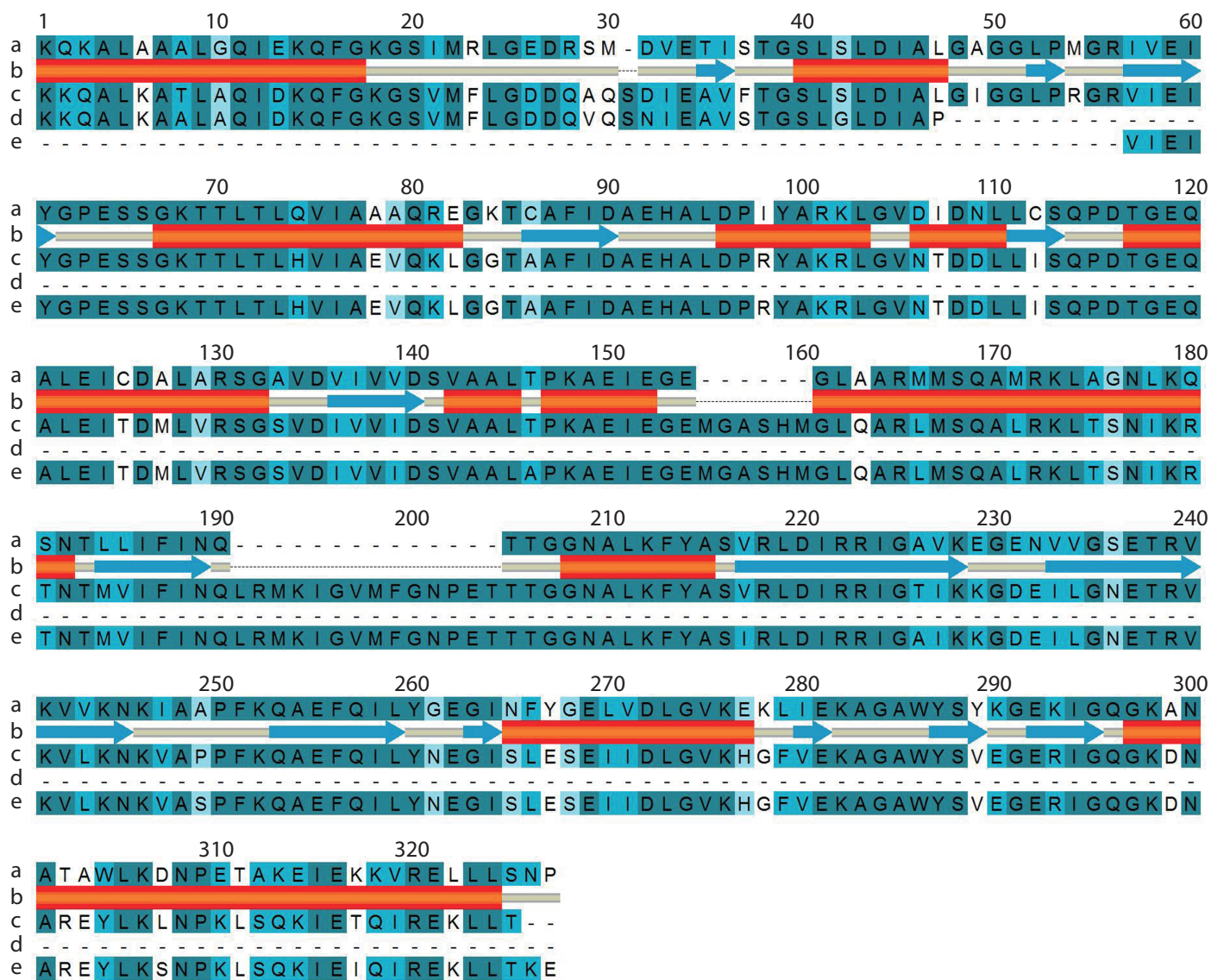

Supplement: Additional file 3 — Figure S3. Part A. 3D homology models reconstructed for RecA of the Calyptogena phaseoliformis symbiont. Homology modeling using the Swiss-Model Workspace (http://swissmodel.expasy.org/) was based on the 3D structure of Escherichia coli RecA (PDB accession number 1U94: [24]) as a template. A, Alignment of amino acid sequences of Calyptogena clam symbiont RecA and E. coli. RecA. Sequences were aligned with ClustalW. a, RecA sequence from Lys-6 to Pro-331 in E. coli; b, secondary structure of E. coli RecA (1U94). Red rectangles, α-helices; blue arrows, β-strands. c, RecA sequence from Lys-5 to Thr-329 in the symbiont of C. phaseoliformis. d, RecA sequence of N-terminal ORF in the symbiont of C. fausta. e, RecA sequence of C-terminal ORF in the symbiont of C. fausta. [file 1471-2148-11-285-S3.PDF]

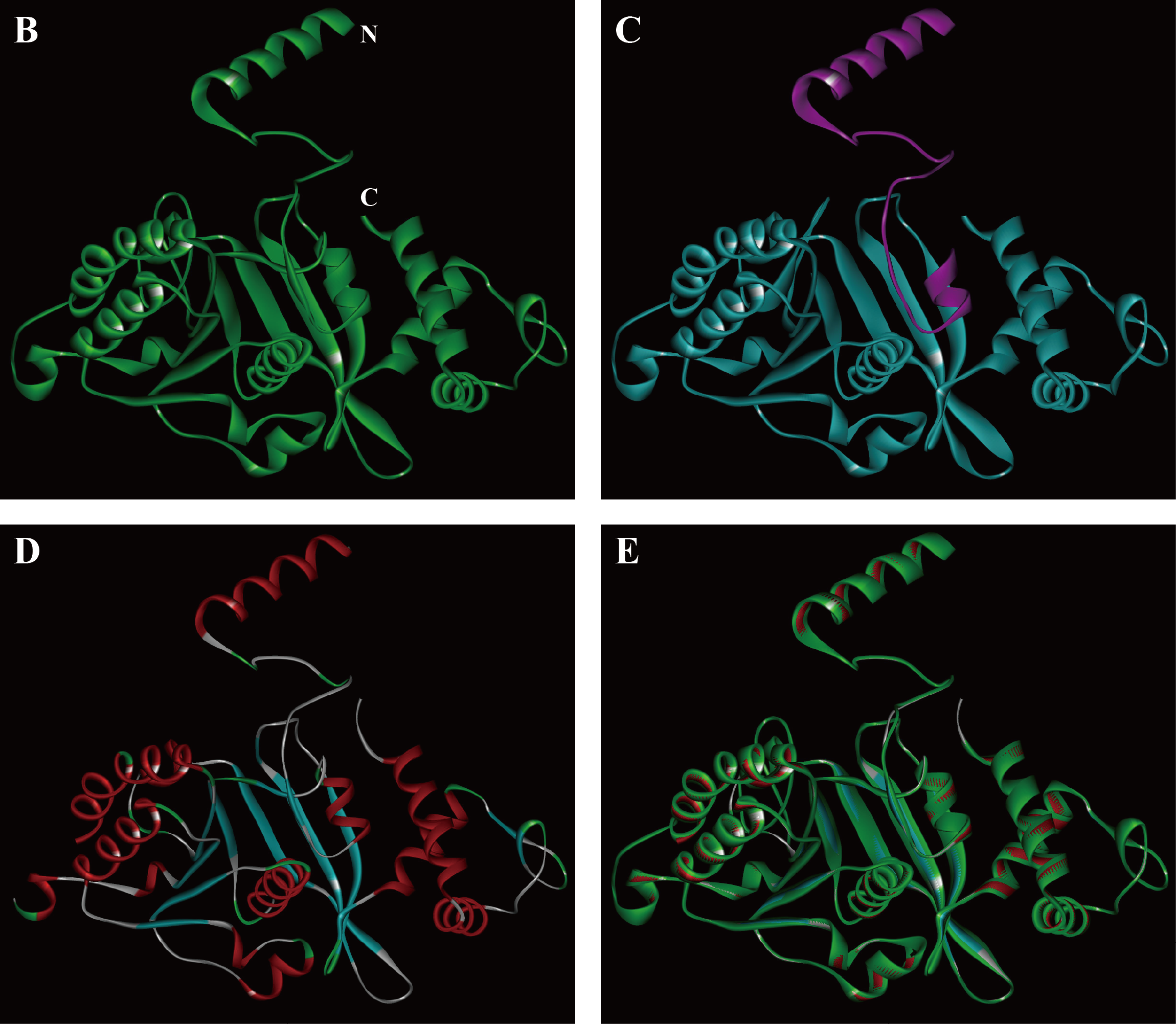

Supplement: Additional file 4 — Figure S3. Parts B-E. 3D homology models reconstructed for RecA of the Calyptogena phaseoliformis symbiont. Homology modeling using the Swiss-Model Workspace (http://swissmodel.expasy.org/) was based on the 3D structure of Escherichia coli RecA (PDB accession number 1U94: [24]) as a template. B, Homology model reconstructed for C. phaeoliformis symbiont RecA. C, Homology model reconstructed for N-terminal and C-terminal amino acid peptides of RecA in C. fausta symbiont. N-terminal and C-terminal peptides are shown in violet and light blue, respectively. D, Crystal structure of E. coli RecA (accession # = 1U94); α-helices and β-sheets are indicated in red-green, and light blue, respectively. E, Merged 3D structures of RecAs of E. coli (D) and of C. phaseoliformis symbionts (B) showing that their 3D structures are nearly the same. This suggests that the C. phaseoliformis symbiont RecA is intact and functional. [file 1471-2148-11-285-S4.PNG]

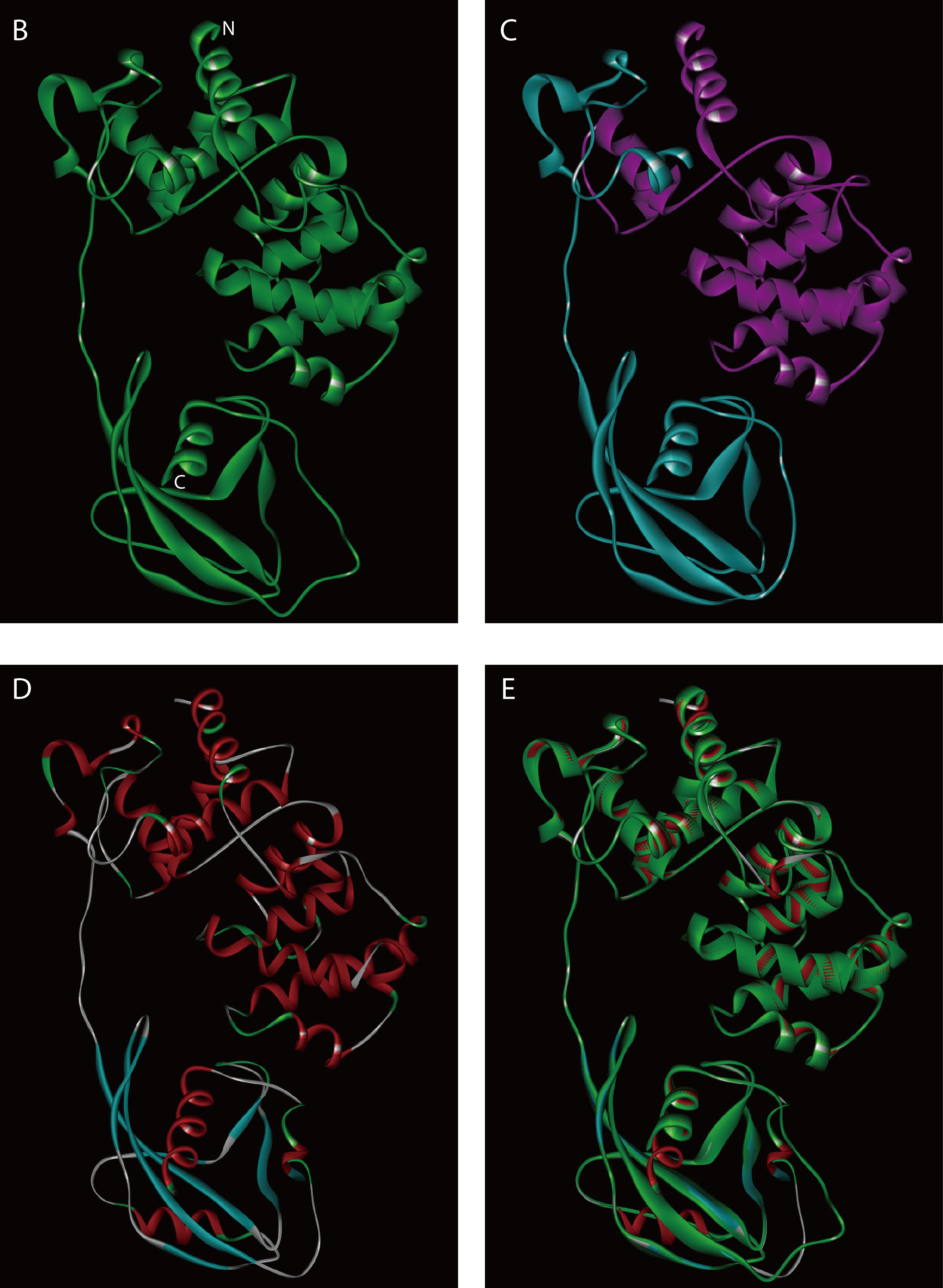

Supplement: Additional file 6 — Figure S4. Parts B-E. 3D homology models reconstructed for MutY of the C. phaseoliformis symbiont. B, Homology model reconstructed for C. phaseoliformis symbiont MutY. C, Homology model reconstructed for N-terminal (violet) and C-terminal (light blue) amino acid peptides of MutY in R. magnifica. D, Crystal structure of G. stearothermophilus MutY (accession # = 3FSP). α-Helices and β-sheets are indicated as red-green and light blue, respectively. E, Merged 3D structures of MutY of G. stearothermophilus (D) and C. phaseoliformis symbionts (B). [file 1471-2148-11-285-S6.PNG]

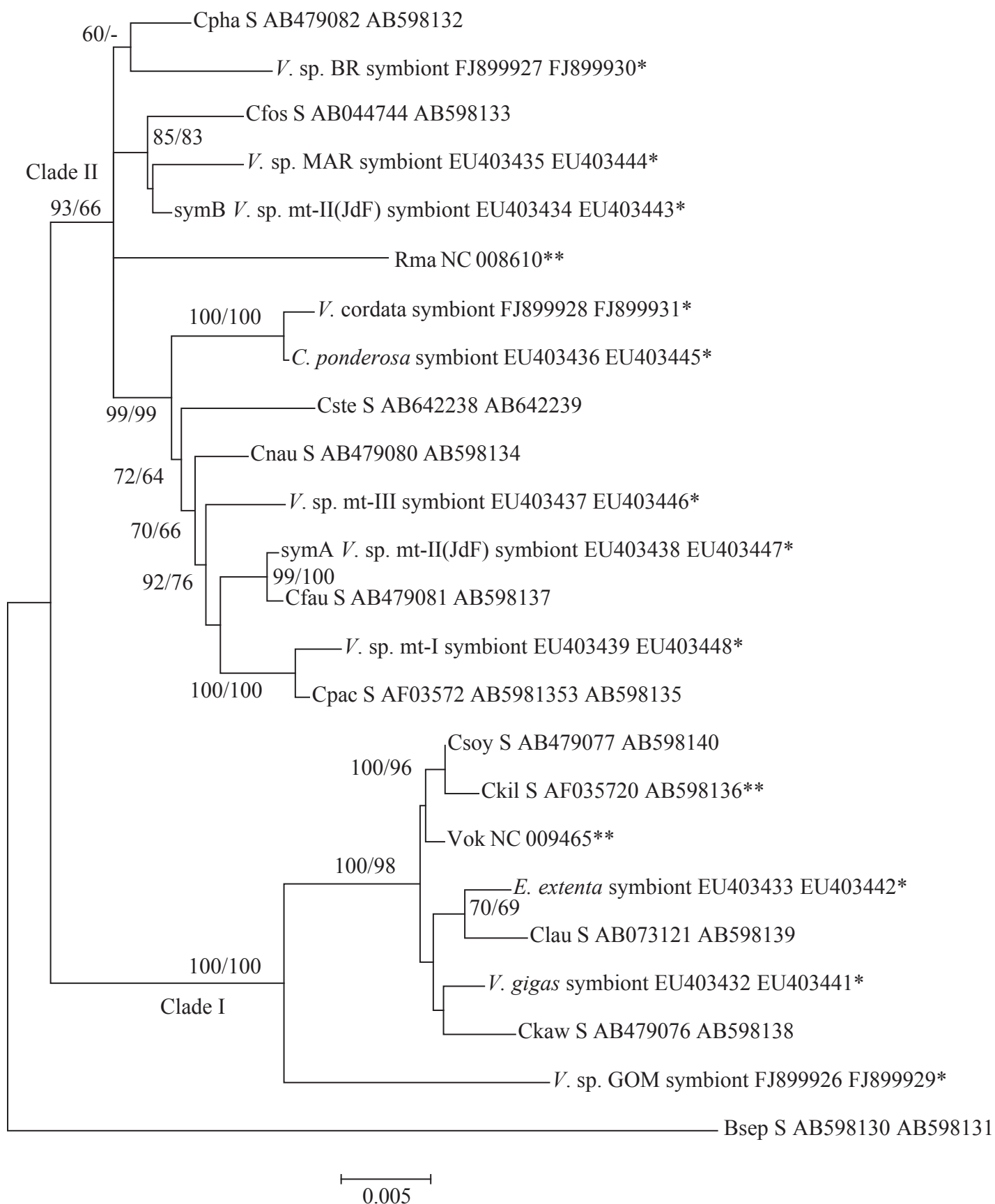

Supplement: Additional file 7 — Figure S5. Phylogenetic tree of the Calyptogena clam symbionts including those of reported in Stewart et al. 2009 [28]. 16S and 23S rRNA gene sequences of the symbionts reported in the present study and of those reported in Stewart et al. (2009) [28] were concatenated and used for phylogenetic tree reconstruction. Topology of the tree constructed using the maximum likelihood method is shown with bootstrap values (> 50%) obtained from the neighbor joining and maximum likelihood methods at each node. Accession numbers of the sequences are shown in the tree. Names and abbreviations of the symbionts are the same as those in Table 3 of the present study or those in [28]. Abbreviations for generic names: C., Calyptogena; E., Ectenagena; V., Vesicomya. *Symbionts reported in Stewart et al. [28]; **symbionts reported in both Stewart et al. [28] and the present study. [file 1471-2148-11-285-S7.PDF]
