# Supplementary material for: Loss of genes for DNA recombination and repair in the reductive genome evolution of thioautotrophic symbionts of Calyptogena clams
Source: BMC Evol Biol. 2011 Oct 3;11:285. doi: 10.1186/1471-2148-11-285 (PMC3202245; doi:10.1186/1471-2148-11-285)
Supplement: Additional file 5 — Figure S4. Part A. 3D homology models reconstructed for MutY of the C. phaseoliformis symbiont. The model was reconstructed using the Swiss-Model Workspace (http://swissmodel.expasy.org/) based on the crystal structure of Geobacillus stearothermophilus MutY [26] (PDB accession # = 3FSP) as a template. A, Alignment of MutY amino acid sequences of Calyptogena symbionts and G. stearothermophilus. Sequences were aligned using ClustalW. a, MutY sequence from Phe-8 to Ser-360 in G. stearothermophilus. b, Secondary structure of G. stearothermophilus MutY (3FSP). Red rectangles, α-helices; blue arrows, β-strands. c, MutY sequence from Val-1 to Asp-341 in the symbiont of C. phaseoliformis. d, MutY sequence of N-terminal ORF in Ruthia magnifica. e, MutY sequence of C-terminal ORF in R. magnifica. [file 1471-2148-11-285-S5.PDF]

A

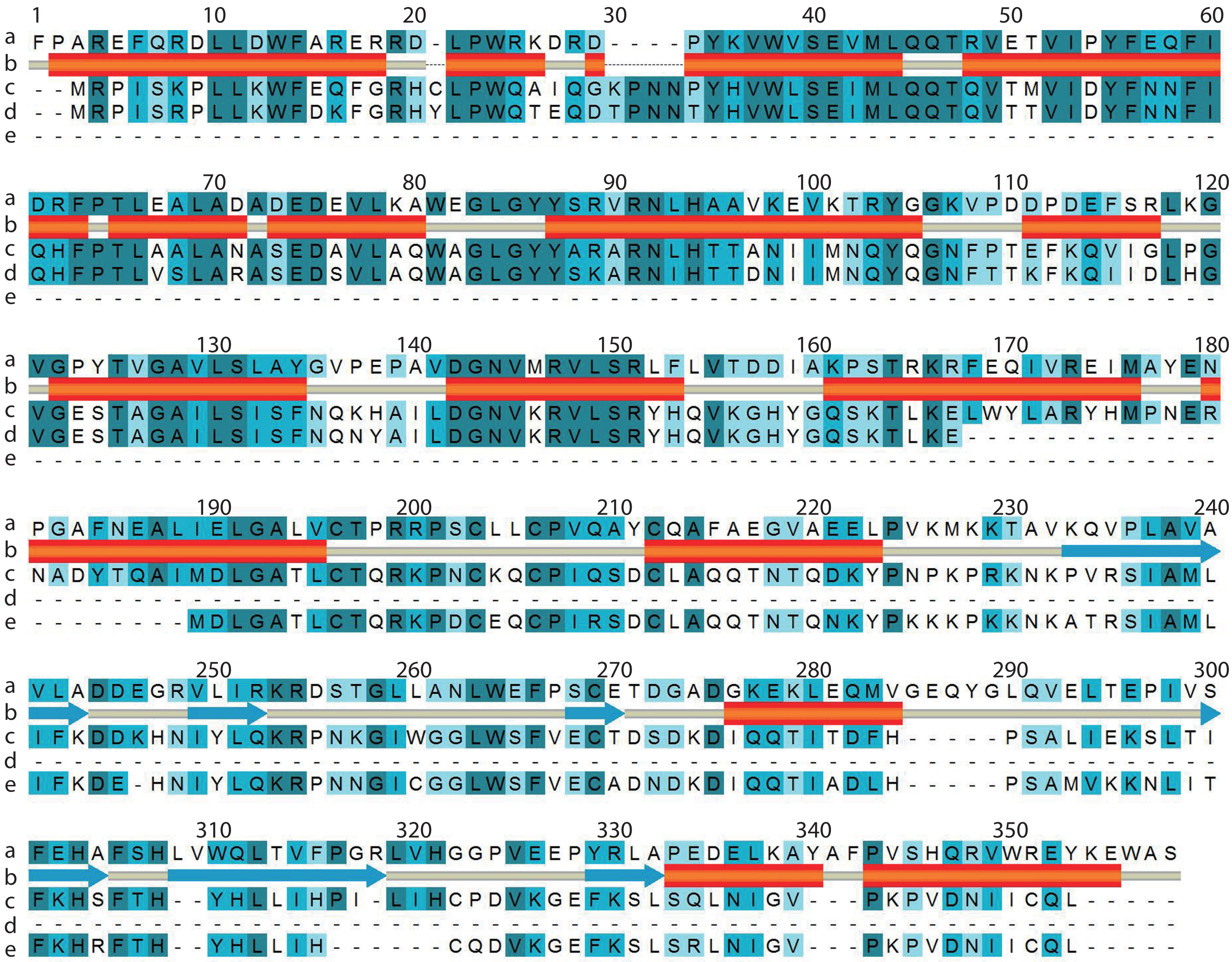
1 10 20 30 40 50 60
a FPAREFQRDLLDWFARERRD-LPWRKDRD----PYKVWVSEVMLQQTRVETVIPYFEQFI
b
c --MRPISKPLLKWFEQFGRHCLPWQAIQGKPNNPYHVWLSEIMLQQTQVTMVIDYFNNFI
d --MRPISRPLLKWFDKFGRHYLPWQTEQDTPNNTYHVWLSEIMLQQTQVTTVIDYFNNFI
e
70 80 90 100 110 120
a DRFPTLEALADADEDEVLKAWEGLGYYSRVRNLHAAVKEVKTRYGGKVPDDPDEFSRLKG
b
c QHFPTLAALANASEDAVLAQWAGLGYYARARNLHTTANIIMNQYQGNFPTEFKQVIGLPG
d QHFPTLVSLARASEDSVLAQWAGLGYYSKARNIHTTDNIIMNQYQGNFTTKFKQIIDLHG
e
130 140 150 160 170 180
a VGPYTVGAVLSLAYGVPEPAVDGNVMRVLSRLFLVTDDIAKPSTRKRFEQIVREIMAYEN
b
c VGESTAGAILSISFNQKHAILDGNVKRVLSRYHQVKGHYGQSKTLKELWYLARYHMPNER
d VGESTAGAILSISFNQNYAILDGNVKRVLSRYHQVKGHYGQSKTLKE
e
190 200 210 220 230 240
a PGAFNEALIELGALVCTPRRPSCLLCPVQAYCQAFAEGVAEELPVKMKKTAVKQVPLAVA
b
c NADYTQAIMDLGATLCTQRKPNCKQCPIQSDCLAQQTNTQDKYPNPKPRKNKPVRSIAML
d
e --------MDLGATLCTQRKPDCEQCPIRSDCLAQQTNTQNKYPKKKPKKNKATRSIAML
250 260 270 280 290 300
a VLADDEGRVLIRKRDSTGLLANLWEFPSCETDGADGKEKLEQMVGEQYGLQVELTEPIVS
b
c IFKDDKHNIYLQKRPNKGIWGGLWSFVECTDSDKDIQQTITDFH-----PSALIEKSLTI
d
e IFKDE-HNIYLQKRPNNGICGGLWSFVECADNDKDIQQTIADLH-----PSAMVKKNLIT
310 320 330 340 350
a FEHAFSHLVWQLTVFPGRLVHGGPVEEPYRLAPEDELKAYAFPVSHQRVWREYKEWAS
b
c FKHSFTH--YHLLIHPI-LIHCPDVKGEFKSLSQLNIGV---PKPVDNIICQL-----
d
e FKHRFTH--YHLLIH------CQDVKGEFKSLSRLNIGV---PKPVDNIICQL-----
